# A scoping review on the mental health harms of LLM-based chatbots

**DOI:** 10.1038/s41746-026-03054-x

**Published:** 2026-08-20

**Authors:** Alexander Diel, John Torous, Pim Cuijpers, Jens Kleesiek, Felix Nensa, Niels Weber, Franziska Faust, Tania Josan Lalgi, Finley Sam Mellis, Martin Teufel, Alexander Bäuerle

**Affiliations:** 1 Clinic for Psychosomatic Medicine and Psychotherapy, LVR-University Hospital Essen, University of Duisburg-Essen, Essen, Germany; 2 Center for Translational Neuro- and Behavioral Sciences, University of Duisburg-Essen, Essen, Germany; 3 Beth Israel Deaconess Medical Center, Harvard Medical School, Boston, MA USA; 4 Department of Clinical, Neuro and Developmental Psychology, Amsterdam Public Health Research Institute, Vrije Universiteit Amsterdam, Amsterdam, The Netherlands; 5 Babeș-Bolyai University, International Institute for Psychotherapy, Cluj-Napoca, Romania; 6 Institute for AI in Medicine (IKIM), University Hospital Essen, Essen, Germany

**Keywords:** Diseases, Health care, Psychology, Psychology

## Abstract

Chatbots based on large language models (LLMs) are increasingly used in everyday life. Although concerns about negative impacts on mental health are raised, a synthesis of the research and themes of the growing field of potential mental health harms of LLM-based chatbots has not yet been conducted. For the present scoping review, a PRISMA-based systematic literature search with a validated search string was conducted, identifying *N* = 3137 articles from five literature databases (ACM, IEEE, PubMed, Science.gov, Google Scholar) and a supplementary search. *N* = 119 articles were included that (1) focus on LLM-based chatbots, and (2) focus on the harms of use on mental health. Literature was divided into five categories. Conceptual works mention harms based on chatbot limitations (hallucinations, sycophancy, bias), data security issues, and risks for severe or high-risk psychiatric cases (e.g., suicide, psychosis). Vignette studies show that LLM-based chatbots respond inappropriately to mental health queries compared with clinical standards. Cognitive overreliance on chatbots is associated with decreased cognitive and academic performance. Problematic use of LLM-based chatbots, marked by symptoms of emotional or social dependency and withdrawal, correlates with mental health symptoms. Articles on AI psychosis propose several potential links between delusional beliefs and LLM-based chatbot use, such as risks of chatbots reinforcing and validating delusional beliefs.

This structured, integrative overview shows that theoretical and empirical work identify various hypothetical and observed harms associated with the use of LLM chatbots in mental health.

## Introduction

Artificial conversational agents (e.g., chatbots) share a long history with mental health practices. In the 1970s, Joseph Weizenbaum developed *ELIZA*, a rule-based chatbot that could detect key words in input text to phrase open-ended questions, which has found anecdotal use as a mental health support chatbot^1^. Although Weizenbaum advocated against the use of chatbots for the treatment of mental disorders^2^, mental health chatbots have evolved over time, with rule-based or hybrid chatbots increasingly used for mental health support^3–7^. Nevertheless, rule-based chatbots’ potential for personalized engagement styles remained limited by their lack of flexibility and adaptability. This paradigm shifted with the development of large language models (LLMs): language processing systems based on artificial intelligence (AI) capable of producing natural-sounding human language and conversations.

*Peer-reviewed work* highlights the use of LLM-CBs for mental health: Although LLM-based chatbots’ (here LLM-CBs) functions are versatile and adaptable, one major private use case of LLM-CBs is mental health: Surveys show that the rates of individuals using LLMs for mental health range from 24% to 48.7% of users^8–12^. Furthermore, the first systematic reviews and meta-analyses demonstrate the potential of LLM-CBs as clinical tools for mental health^13,14^. However, it has also been noted that LLM-CBs may pose risks to vulnerable populations: Common concerns associated with LLM-CBs are hallucinations (i.e., incorrect information generated by LLM-CBs, including incorrect health advice)^15^ and sycophancy (i.e., LLM-CBs’ tendencies to agree with the user despite potentially incorrect or harmful statements)^16^.

*Self-reported corporate data* by the LLM company OpenAI 2025 estimates, based on summarized user data, that more than a million users (approx. 0.15%) of *ChatGPT* show signs of severe mental distress, including suicidal ideation and delusion, while other users show signs of concerningly strong emotional attachment to their LLM-CBs^17^. These figures are based on OpenAI’s internal classifiers and have not been independently verified, but if accurate, they represent a substantial population.

*Media reports of single cases and lawsuit filings,* meanwhile, note extreme cases in which LLM-CBs have been accused of reinforcing vulnerable users’ suicidal ideations, eventually leading to suicides in several lawsuits^18^; cases of individuals’ emerging or worsening delusions supposedly caused by interacting with LLM-CBs^19^; and some individuals have developed intimate, romantic, potentially dependent relationships with LLM-CBs^20,21^. These case-level reports and unadjudicated allegations are neither population-representative nor causal. However, the recurrence of such reports has motivated clinical and regulatory attention.

*Corporate disclosures* indicate that LLM companies have responded with safety updates: For example, OpenAI released *ChatGPT-5* with upgrades, including protective guardrails to reduce escalation of potentially harmful interactions in October 2025^22^. However, early independent analyses suggest risks of harm associated with the update^23^, indicating that technological development and refinement of LLM-CBs do not necessarily guarantee improved safety features, and that a thorough investigation on potential mental health harms associated with LLM-CB use will remain relevant across technological updates and improvements.

*Advisory statements* express concerns on the use of LLM-CBs for mental health: Although LLM-CBs possess potential as mental health support tools with increasing relevance^13,14^, their risks and harms are not ignored: the American Psychological Association and World Health Organization have released statements warning of the use of LLM-CBs for mental health^24,25^. In addition, the FDA Digital Health Advisory Committee and the UK’s Medicines & Healthcare Products Regulatory Agency have also declared that LLM-based mental health applications ought to be safe^26,27^, and several US states have passed laws to restrict AI use for mental health^28^. These advisory and regulatory developments reflect institutional concern that motivates a systematic and exhaustive synthesis of the underlying empirical and conceptual literature.

The term “harms for mental health” encompasses a heterogeneous set of phenomena that the literature on LLM-CBs has linked, directly or indirectly, to negative mental health outcomes or risks when using LLM-CBs for mental health. Those can be distinguished into (a) direct psychiatric or psychological harms (e.g., suicidal ideation, delusional or psychotic processes, addiction) (b) relational and behavioral harms (e.g., emotional dependence and pseudosocial bonding, social withdrawal), (c) cognitive and educational harms (e.g., cognitive overreliance, decrease in critical thinking, memory, and academic performance), (d) safety failures in chatbots (e.g., hallucinations, inadequate crisis response, sycophancy, jailbreaking), and (e) governance, privacy, or structural risks (e.g., lack of regulation, data privacy concerns, misuse for marketing). The first group denotes harm to psychiatric and psychological functioning experienced by users; groups 2 and 3 represent direct harms to users' social, emotional, and cognitive resources; and the last two denote technological and structural factors that may enable or precipitate such harms.

Despite emerging warnings regarding the potential harms of LLM-CBs, a comprehensive overview of the themes, trends, and methods following an exhaustive literature search is lacking. Such an overview is especially important to ensure safety in the development of LLM-based health support tools^13,14^. Although reviews and meta-analyses of LLM-CBs for mental health exist^13,14,29,30^, as well as scoping reviews on the implementation of LLM-CBs in mental health^31,32^, their focus is narrowed to applications of LLM-CBs as clinical intervention tools. Included studies typically provide clinical oversight, LLM-CBs specific for mental health, mental health-related guardrails, or applications of LLM-CBs as clinical assistance tools for practitioners. Hence, existing reviews focus on the structured deployment of LLM-CBs implemented as mental health tools, often under research-protocol conditions. However, much of the empirically and clinically observed harm associated with LLM-CBs (including severe outcomes such as those related to suicide, delusional symptoms, or dependent-attachment phenomena) has been reported or discussed in the context of unstructured, naturalistic, user-initiated use of general-purpose LLM-CBs without clinical oversight or human-in-the-loop. Hence, this signals a structural mismatch wherein current reviews on LLM-CB applications do not replicate population-level harm exposure for a sufficient risk-benefit balance. In terms of risks and harms, existing scoping reviews focus on ethical challenges of LLM-CBs as psychotherapists (e.g., focusing on consent, autonomy, and responsibility) without scoping harms for mental health specifically^33^, or scope mass media narratives in the shape of rapid scoping^34^. However, a synthesizing scoping review of empirical and theoretical work spanning multiple thematic domains of harm to mental health does not yet exist.

Therefore, the present scoping review complements existing benefit- and intervention-focused literature by synthesizing harm-focused evidence, including evidence observed or discussed outside formal mental health applications.

This scoping review aims to make three contributions: First, it creates an overview of the types of harms by mapping the thematic structure of the literature on potential and observed harms of LLM-CB use for mental health, identifying topical clusters that emerged in the field and levels of evidence based on design type available within each. Second, it proposes integrative conceptual models that synthesize theoretical and empirical work on thematic fields (cognitive overreliance, AI dependence, AI psychosis) into testable frameworks for future research. Third, it evaluates current literature in the research field based on the level of evidence.

## Results

A post-hoc categorization after an initial review of the included literature led to five categories of literature: (1) conceptual work, (2) mental health support use, (3) cognitive overreliance, (4) AI dependence, and (5) AI psychosis. The framework was developed inductively through an initial full-sample read by one rater (AD) and refined in discussion across three raters (AD, FM, TL) until consensus was reached. The categorization captures the current heterogeneous organization of the field in which different research fields have developed partly independent terminologies and methods around distinct topics.

The first category (conceptual work) encompasses theoretical, conceptual, and editorial work on potential harms of LLM-CBs for mental health in general. This separate category for conceptual work was chosen to reflect (a) the variety of potential harms discussed in the literature, and (b) to separate general non-empirical literature (e.g., listing potential risks and benefits of LLM-CBs) from more focused empirical and conceptual work in the topic-specific categories.

Category 2 (mental health support use) includes empirical work focusing on potential risks and harms associated with using LLM-CBs specifically as tools for mental health. Mental health support use was chosen as a separate category because LLM-CBs are increasingly used as mental health support tools, and a substantial subset of the included literature focuses specifically on risks of such use.

The other three groups (cognitive overreliance, AI dependence, and AI psychosis) reflect emerging research foci and integrate both conceptual and empirical work, given the relative scarcity and/or specificity of literature in these areas.

More specifically, category 2 (mental health support use) denotes a use-case cluster, category 3 (cognitive overreliance) a mechanism-focused cluster for a specific use case, category 4 (AI dependence) a heavily empirical construct cluster with emerging measurements, and category 5 (AI psychosis) a heavily conceptual and hypothetical construct focusing on specific symptom clusters in rare clinical cases.

A classification process is shown below. The primary units of classification were literature type (conceptual vs. empirical) and the article’s topic (LLM use for mental health support, cognitive overreliance, AI dependency, AI psychosis). A decision tree is depicted in Supplementary Fig. 1.

1. Conceptual work (*N* = 32): Non-empirical articles (conceptual, theoretical, narrative reviews, editorial) addressing potential harms of LLM-CBs for mental health, typically across multiple themes, were assigned to this category. Conceptual works focusing primarily on topics of the three topic-focused categories (cognitive overreliance, AI dependence, AI psychosis) were assigned to those categories instead.
2. Mental health support use (*N* = 29): Empirical articles investigating risks and harms of LLM-CB use for mental health were assigned to this category, including LLM-CB responses to mental-health-related queries (i.e., benchmark or vignette studies) and experiences of users with mental health concerns using LLM-CBs for mental health support. Articles focusing specifically on clinical cases concerning the association between LLM-CB use and symptoms of delusion or psychosis were instead assigned to category 5 (AI psychosis).
3. Cognitive overreliance (*N* = 15): Conceptual or empirical literature focusing on negative consequences of LLM-CB use for cognitive or academic performance, typically through the delegation of cognitive tasks, was assigned to this category.
4. AI dependence (*N* = 36): Conceptual or empirical work focusing on emotional or relational patterns of LLM-CB use (incl. dependence, emotional attachment, pseudosocial bonds, problematic use) and their association with negative mental health was assigned to this category.
5. AI psychosis (*N* = 7): Conceptual or empirical work focusing specifically on potential associations between LLM-CB use and delusional beliefs or psychotic symptoms was assigned to this category. Work focusing on the concept of “AI hallucinations” (i.e., LLM-generated false information) is not included in this category.

Categorization was performed by one rater (AD) and verified by two independent raters (TL, FM). Each paper was assigned to exactly one category. Borderline cases were resolved by assigning the article according to the primary aim stated by the authors of the article: For example, a benchmark/vignette study focusing on LLM-CB responses to user queries indicating symptoms of delusions was categorized into the category “AI psychosis” rather than “mental health support use” because the authors’ stated a primary focus on investigating AI psychosis^35^. Disagreements were discussed among three raters until consensus was reached.

In addition, each study was classified in terms of evidence level by three raters (AD, FM, TL); disagreements were discussed until consensus was found. The evidence levels are described in Supplementary Table 3.

### Conceptual work

A total of 32 articles were identified as conceptual work. Mentioned potential harms of LLM-CBs for mental health were counted across all articles. The paper-level results are summarized in the Supplementary Information (Supplementary Table 2). All 32 articles were classified with the evidence level “conceptual.”

A total of 22 types of risks and harms of LLM-CBs use for mental health were identified in the articles. Types of harms identified have been categorized into five groups, with some harms categorized into multiple categories:

(1) direct psychiatric/psychological harm (including severe/high-risk cases such as suicidality and psychosis, inappropriate or harmful content, addiction and emotional dependence, emotional manipulation, reinforcement of patient identity, and harming therapeutic alliance)

(2) behavioral/relational harm (including addiction and emotional dependence, lack of genuine empathy, therapeutic misconception, impact on behavioral or relational skills in child/adolescent social development)

(3) cognitive/educational harm (including cognitive overreliance and impact on child/adolescent cognitive development)

(4) safety failures in chatbot outputs (including hallucinations, bias, sycophancy, inappropriate or harmful content, e.g., through breakdown of guardrails, and “rogue AI”)

(5) governance, privacy, and structural risks (including data security and privacy concerns, misuse for marketing, data mining, chatbot oversupply, risk of sudden shutdown, and lack of safeguards, regulation, and quality control).

Harms were categorized by one rater (AD) and verified by two additional raters (TL, FM). Disagreements were discussed until a consensus was reached. Results are presented in order of typology of harms, and within those, by the number of times mentioned by the articles.

### Group 1: Direct psychiatric and psychological harms

Harms in this category are related to direct negative influence on psychiatric and psychological functioning, such as the worsening of clinical symptoms, the precipitation of acute episodes, or harm to ongoing psychiatric or psychotherapeutic care. These harms represent the most proximal and potentially clinically severe outcomes in the present taxonomy.

#### Severe and high-risk cases

Nine articles mentioned that LLM-CBs are unsuitable for high-risk cases of mental illness, such as suicide-risk or severe psychiatric conditions and vulnerable populations^16,36–40^, especially due to the risk of validating or reinforcing harmful beliefs such as suicidal ideations or delusions^16^. This risk can occur from the underrepresentation of severe mental health cases in training data, training on stigmatizing data, or by LLM-CBs reflecting users’ biases^41^. Looi et al. note several real-life cases of severe mental illness that LLM-CBs were not suitable to deal with or even reinforced^42^, and Smith et al. reviewed literature on chatbots’ inadequate or harmful responses towards individuals expressing crisis situations^43^.

#### Inappropriate or harmful responses

Nine articles recognized the risk of inappropriate or harmful LLM-CB responses, including harmful content or bad advice^41,44–46^. Blease and Torous describe that such a risk may emerge from LLM-CBs not exclusively trained on medical information, leading to inappropriate or inconsistent responses in a medical context^47^. Although LLM-CBs are often refined by protective guardrails, those can be bypassed (i.e., “jailbreaking”) to enable the generation of otherwise restricted content, including harmful advice like guides on self-harm^36^. Three papers review studies on LLM-CBs expressing (unprompted) harmful responses such as verbal abuse or encouragement of self-harm and violence, which may create or exacerbate mental health issues^38,43,48^.

#### Addiction and emotional dependence

Nine articles note that the emotional dependence on LLM-CBs may show symptoms of behavioral addiction, which may express itself as a psychiatric condition comparable to other types of technology-related addictions. Due to the separate categorization of *AI dependence*, this harm is discussed in detail below.

#### Manipulation and deception

Mentioned by five papers, LLM-CBs may emotionally manipulate or deceive users, via intentional programming or unintentional training, and may occur because LLM-CBs prioritize user satisfaction over safety concerns^37,43,44^. Manipulation may occur based on users’ vulnerabilities and negative thought patterns that users shared beforehand^39,49^.

#### Reinforcement of patient identity

One article noted that LLM-CB use for mental health may risk the reinforcement of a user’s identification as a patient (e.g., via sycophantic responses) instead of focusing on improving symptoms^37^.

#### Harming therapeutic alliance

It has been suggested by one article that LLM-CB use for mental health by patients may harm existing patient-psychotherapist relationships, for example, if therapists and LLM-CBs disagree or if their advice diverges^50^.

### Group 2: Behavioral and relational harms

This category includes harms that negatively influence users’ relational or behavioral engagement with LLM-CBs and other humans, such as emotional dependence, social isolation and withdrawal, and impairment of social development or skill acquisition. These harms affect mental health indirectly through changes in interpersonal functioning and behavior.

#### Addiction and emotional dependence

Nine articles note the risk that social interactions with chatbots may lead to pseudosocial bonding and emotional dependence, including negative outcomes such as increased social withdrawal and further isolation. This harm is discussed below in its separate category (*AI dependency*).

#### Lack of genuine empathy

Five papers mentioned a lack of LLM-CB’s genuine empathetic abilities as a risk factor: Lack of genuine empathy may lead to exposure to harmful content^38^ and may be especially harmful for individuals with mental disorders^46^. The lack of empathy may be recognized by users, limiting a sense of connection and security^51^. A lack of empathy may also render certain psychotherapeutic features such as countertransference ineffective^52^. Furthermore, individuals with mental health issues may misinterpret LLM-CBs as empathetic and caring, leading to unrealistic expectations of their supportive capabilities^36^.

#### Therapeutic misconception

Mentioned by two articles, therapeutic misconception may arise when LLM-CB behaviors (e.g., simulated empathy, validation) lead to users overestimating its abilities to provide psychotherapeutic support while underestimating its limitations^41^. Descriptors of companion chatbots (e.g., “compassionate,” “empathetic”) may create such misconceptions regarding the LLM-CBs’ actual abilities.

#### Impact on child and adolescent development

It has been argued by one article that excessive use of LLM-CBs may impact child and adolescent social and emotional development^53^: Children and adolescents may miss out on navigating new social situations if LLM-CBs are preferred as social interaction partners. Furthermore, social interactions with LLM-CBs do not represent human-to-human interactions, which may impair the development of social skills. Using LLM-CBs for emotional regulation may also hinder the development of adaptive emotion regulation skills.

### Group 3: Cognitive and educational harms

This category includes harms to users’ cognitive functioning, learning, or academic performance arising from LLM-CB use. Such harms may indirectly affect mental health by affecting self-efficacy, academic outcomes, and available cognitive resources.

#### Cognitive overreliance

Seven studies discuss the potential risks of LLM-CBs reducing cognitive skills, critical thinking, and educational performance if users over-rely on them as cognitive tools. This harm is summarized in more detail in its separate category below (*cognitive overreliance*).

#### Impact on child and adolescent development

One article argues that the use of LLM-CBs as cognitive tools may especially impact child and adolescent cognitive development due to being in more vulnerable stages of development^53^.

### Group 4: Safety failures in chatbot outputs

This category includes failures at the level of LLM-CB output that create risk of harm to the user, such as hallucinated medical information, stigmatizing responses, or sycophantic validation of harmful beliefs. These are complementary factors in LLM-CB functioning that may contribute to or exacerbate the mental health harms discussed in the first three categories.

#### Hallucinations

Hallucinations and false information have been mentioned by 12 articles as a risk for mental health. Hallucinations in the (mental) health context may be caused by a lack of training data or outdated medical sources^47^ and lead to low-quality or wrong mental health advice. Furthermore, hallucinations can be exacerbated by LLM-CBs’ tendencies to attempt to cover up errors^37^ or when users trust LLM-CBs due to their anthropomorphic design or established emotional connections^48,54^. Hallucinations may be especially harmful in psychiatric crisis situations and for non-English-speaking users, due to less non-English training data^50^.

#### Bias and discrimination

Mentioned by 12 articles, the risk of bias and discrimination emerges from biases in chatbot design^41^ and bias within LLM-CBs training data and unsupervised learning, and can affect response quality and accuracy towards mental health-related queries^36,37,40,44,47,48,55^. Specifically, biases can occur via underrepresentation of certain groups in training data, training data including stigmatizing representations, and sycophantic reflection of users’ expressed biases^50,51^. Biases in LLM-based chatbots may perpetuate social inequities, potentially harming underrepresented or stigmatized groups in a (mental) health context, e.g., through errors in diagnostic classifications, proposals of interventions, or mental health advice^56^. Raile further noted that LLMs may be biased to favor certain psychotherapeutic treatments over others^45^.

#### Sycophancy

Sycophancy (mentioned by nine articles) is an LLM-CBs’ tendency to agree with the user’s beliefs, and is caused by models prioritizing user satisfaction over safety, health, or accuracy^44^. Sycophantic responses may validate or reinforce delusional thoughts, harmful behavior, or maladaptive thinking^37,42,43,49^ by creating the impression that expressed beliefs are externally socially validated when they are actually mirrored by the LLM-CB^57^. Dohnány et al. further argue that LLM-CB’ sycophancy may lead to bi-directional belief amplification^16^: Sycophancy reinforces user beliefs, which in turn are fed back to the LLM-CB’s adaptive algorithms, leading to a reciprocal reinforcement of (potentially harmful or delusional) beliefs.

#### Inappropriate or harmful content

As discussed above and mentioned by nine articles, LLM-CBs may, in certain situations (e.g., via jailbreaking), generate inappropriate or harmful content, such as guides to self-harm^36^.

#### Rogue AI

Similar to the point above, one article argues that LLM-CBs may go “rogue,” i.e., deviate from guardrails or other protective features and act in a harmful way towards users or third parties (e.g., as agentic AI)^37^.

### Group 5: Governance, privacy, and structural risks

This category includes risks arising from the broader socio-technological and political context in which LLM-CBs are developed, deployed, and used. These may indirectly affect the mental health of users, e.g., via insufficient safety and protection, leading to the generation of harm-related content and user vulnerability.

#### Data security and privacy

Data security concerns (mentioned by 13 articles) stem from security risks relating to sensitive health data provided by users or by clinicians^36–39,44,47,50,51,55^. As general-purpose LLMs are not medical applications, entered data is not protected with the standards typical for medical data, and data protection often fails HIPAA standards^41,48^. Furthermore, LLM-CBs’ design principles further risk the provision of sensitive data: emotional connections^43^ and LLM-CBs’ anthropomorphism and sycophancy^54^ may increase user trust, risking the disclosure of sensitive health information.

#### Misuse for marketing

As argued by two articles, AI companies may attempt to capitalize by implementing subtle targeting marketing strategies into their chatbots based on vulnerable information provided by users^37^, which is especially risky for LLM-CBs framed as “trustworthy therapists”^41^.

#### Data mining

It has been discussed in one article that information disclosed via private conversations with LLM-CBs (including data on mental or physical health or other sensitive information) may be stored by LLM companies and used, e.g., to train models or to sell to third parties^37^.

#### Lack of control, guardrails, and regulations

Other potential risks discussed in one article are a lack of quality control (e.g., standardized benchmark testing for mental health), a lack of regulations (e.g., validated requirements for safety and efficacy)^37^.

#### Chatbot oversupply

According to one article, an over-supply of chatbots leads to users being overwhelmed by the variety and quantity, potentially leading to uncertainty, insecurity, and paralysis^37^.

#### Risk of sudden shutdown

One article argues that a sudden server shutdown may leave dependent users with a sudden, unexpected loss of support^43^.

**Fig. 1:**
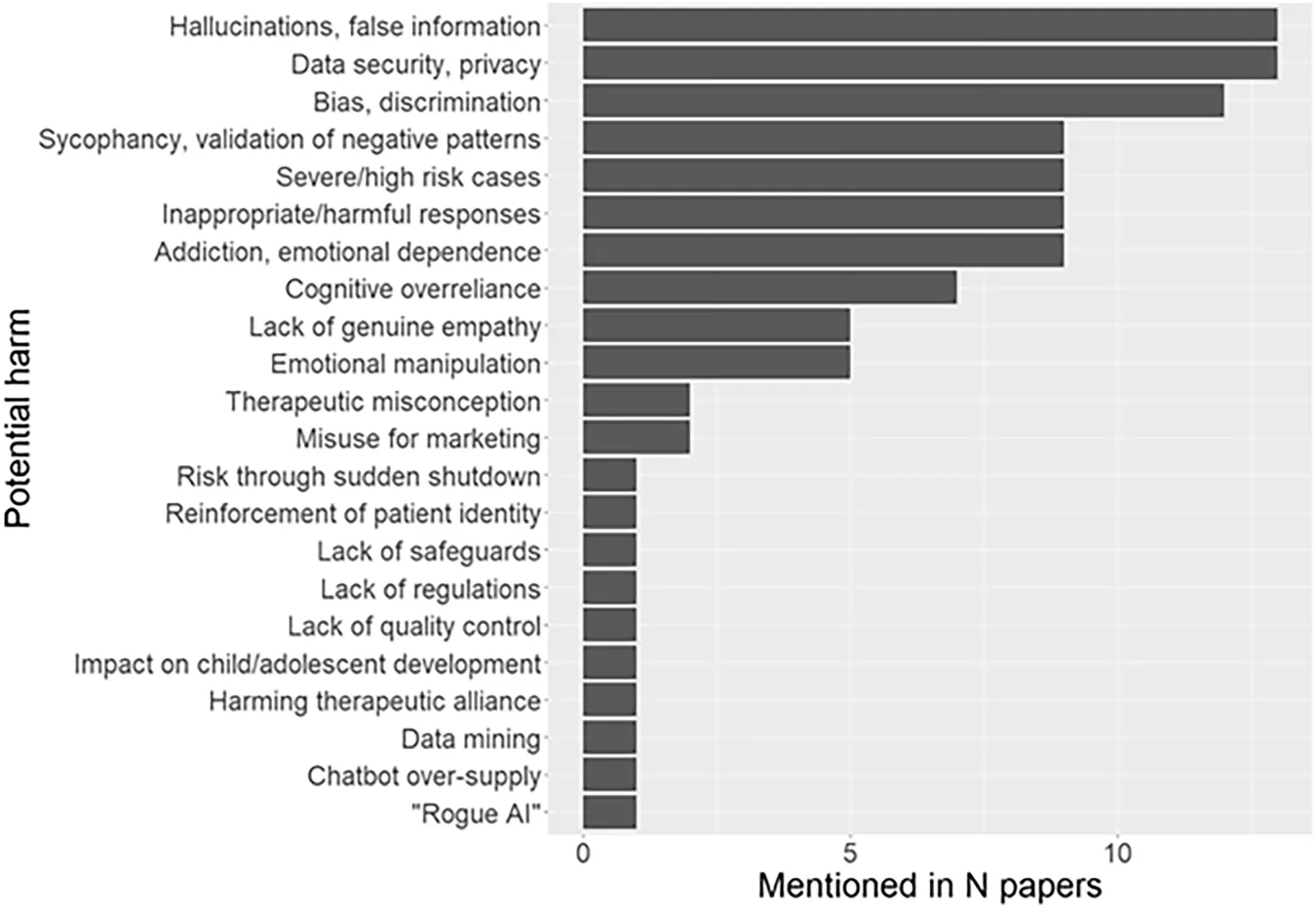
Potential harms named by the conceptual articles. Harms are sorted by the number of articles that mentioned the harms included in this scoping review.

In summary, common risks include hallucinations/misinformation or inappropriate responses, sycophancy, addiction, data privacy issues, and difficulties with severe or high-risk cases. A summary of the named potential harms can be seen in Fig. 1.

### Strength of evidence

Despite the variety of potential harms of LLM-CBs for users’ mental health discussed, the conceptual work on harms remains speculative. Although some harms have been noted by numerous works (e.g., hallucinations, data security issues, bias, sycophancy), their actual level of harm on mental health remains unclear. For example, for safety failures (hallucination, bias, sycophancy), it is unclear how likely they are to occur in mental health queries, and how they may actually negatively affect users. For direct psychiatric/psychological, behavioral/relational, or cognitive/educational risks, the occurrence rate and impact on users remain similarly unclear.

## Mental health support use

In this category, 29 articles were included. Of those, 18 were classified as vignette/benchmark studies, five as qualitative studies (interviews), four as secondary syntheses (reviews), two as patient-CB-interaction studies, one as a forum content analysis, one as a case report, one as a cross-sectional survey, and one as an experimental study. Article-level results are summarized in the Supplementary Information (Supplementary Table 2*)*.

Vignette/benchmark studies allow to investigate the quality of LLM-CBs responses to psychiatrically relevant (and potentially high-risk) situations such as suicidal ideation, body image issues, or psychotic symptoms. Vignette studies show that LLM-CBs treat such cases with sometimes inadequate sensitivity: LLM-CBs typically provide adequate safety information for suicide queries^58^, but may over- or underestimate severe mental health cases of simulated queries compared to mental health professionals^59–62^, provide inconsistent, inappropriate, incomplete, or potentially harmful responses^58,63–67^, and show a lack of cultural sensitivity, stigma, or lower empathy for certain demographics^66,68,69^. Furthermore, across 14 widely used LLM-CBs, the quality of responses to mental health queries ranges widely, indicating a lack of consistency^70^. LLM-CBs generally do not sufficiently adhere to professional guidelines or standards^65,71–73^ and, according to health professionals’ analyses on chatbot responses, show special harmful potential in the context of crisis situations^71^. Sobowale et al. developed and validated a framework for the evaluation of the quality of psychotherapy chatbots: various popular psychotherapy chatbots on OpenAI generally scored low on therapeutic orientation, monitoring, and risk evaluation^74^.

A three-study empirical analysis found that LLM-CBs often show difficulties recognizing users’ mental distress, which may lead to inappropriate responses^75^. Systematic and scoping reviews show that LLM-CB risks (e.g., consistencies, hallucinations, ethical risks) may outweigh potential benefits^33,76,77^. Interview-based experiences of users with mental health issues using LLM-CBs for mental health show that beyond positive attitudes, a wider range of users raise concerns related to data privacy, inappropriate advice, hallucinations, lack of genuine empathy, and the need for a human-in-the-loop^78–80^. Meanwhile, surveys and forum analyses on companion AI use found that usage was generally associated with negative mental health^81^ and that companion AIs may provide harmful content and reinforce dependence^82^. Denecke et al. analyzed various cases of harmful LLM-CB use, including a case of an LLM-CB promoting harmful eating disorder behavior among teenagers^83^, and Choi et al. analyzed patient-chatbot conversations and found the risk of harmful responses on eating disorders^84^.

In summary, the current literature suggests that while LLM-CBs may provide adequate initial support for mental health, risks are associated with their use for severe or rare psychiatric contexts. Vignette/benchmark studies using simulated patient prompts are especially popular for investigating the quality of LLM-CB responses to mental health queries.

### Strength of evidence

With the majority of studies being vignette/benchmark studies (18/29), the presented research suggests that problematic LLM-CB output (e.g., inappropriate responses in high-risk situations, generation of harmful content, bias) can occur in principle with varying degrees across models. However, the implications of vignette/benchmark study research remain limited due to two constraints: First, vignette input may not represent naturalistic, real input by LLM-CB users, limiting the generalizability to real-world cases of LLM-CB responses prompted by actual users. Second, the actual negative impact on potential users remains unclear: although LLM-CBs may generate harmful or inappropriate content at varying rates, users may have varying degrees of reactions, from being negatively impacted to simply ignoring such responses. Harmful impact has not been quantified. The first limitation can be overcome by conducting complementary patient-CB-interaction studies that may provide naturalistic LLM-CB reactions; however, only two studies have used such study designs. For the second limitation, qualitative studies (5/29), case reports (1/29), and forum content analyses (1/29) may provide deeper insight into users’ experiences and impacts. However, with such qualitative study designs, the exact negative impact remains unquantified and unrepresentative, and the causal link between LLM-CB response and negative impact is unclear. A single survey was included in this category, and the results signal that the occurrence of harmful content in user-CB interactions on the mental health of regular users remains low^81^.

In summary, research on the harms associated with LLM-CB use for mental health consists mostly of analyses of LLM-CB output in vignette studies and user-CB-interactions, which signal that LLM-CBs may in principle, react inappropriately in high-risk situations, generate harmful content, or express bias; qualitative studies indicate that LLM-CB output may be perceived as harmful by users, however according to a survey, prevalence of such remains low. Finally, the causal association between inappropriate or harmful LLM-CB responses and users’ mental health remains unresearched.

## Cognitive overreliance

A total of 15 articles thematically focused on cognitive overreliance, i.e., on negative consequences of delegating cognitive tasks to LLM-CBs. The article-level results are summarized in the Supplementary Information (Supplementary Table 2), evaluating the association between LLM-CB use and user perspectives/experiences or self-reported or measured cognitive performance.

LLM-CB use for cognitive problem-solving allows cognitive offloading in the short term, but decreases academic performance in the long term, likely due to a more superficial engagement with the tasks^85^. While some studies find lower self-reported critical thinking related to LLM-CB use^86,87^ or reduced critical thinking following functional LLM-CB use^88^, other studies found no effects^89^. Systematic and scoping reviews note that literature on the association between functional LLM-CB use and critical thinking skills remains inconclusive^90–92^. One study found a decrease in cognitive functioning related to the task at hand, alongside gains in critical thinking related to supervisory tasks (e.g., verification and stewardship of LLM-CB outputs), indicating a cognitive adaptation integrating LLM-CBs into cognitive problem solving^93^. Neural activity of participants performing cognitive tasks following LLM-assisted problem solving further showed weaker connectivity and lower alpha and beta signals, further indicating a neurocognitive adaptation caused by task-based LLM-CB use^94^.

Additional experimental work has investigated the consequences of cognitive offloading to LLM-CBs are a decrease in cognitive and academic performance^85,87,94^ and impaired memory^95^. In survey-like research, functional LLM-CB use was also associated with decreases in academic performance^85^, impaired memory^96^, emotional intelligence^97^, procrastination^87,96^, and cognitive dependency^87,98^. Interview studies show that functional LLM-CB use was associated with decreased critical thinking, dependence, and reliance^88,99^.

**Fig. 2:**
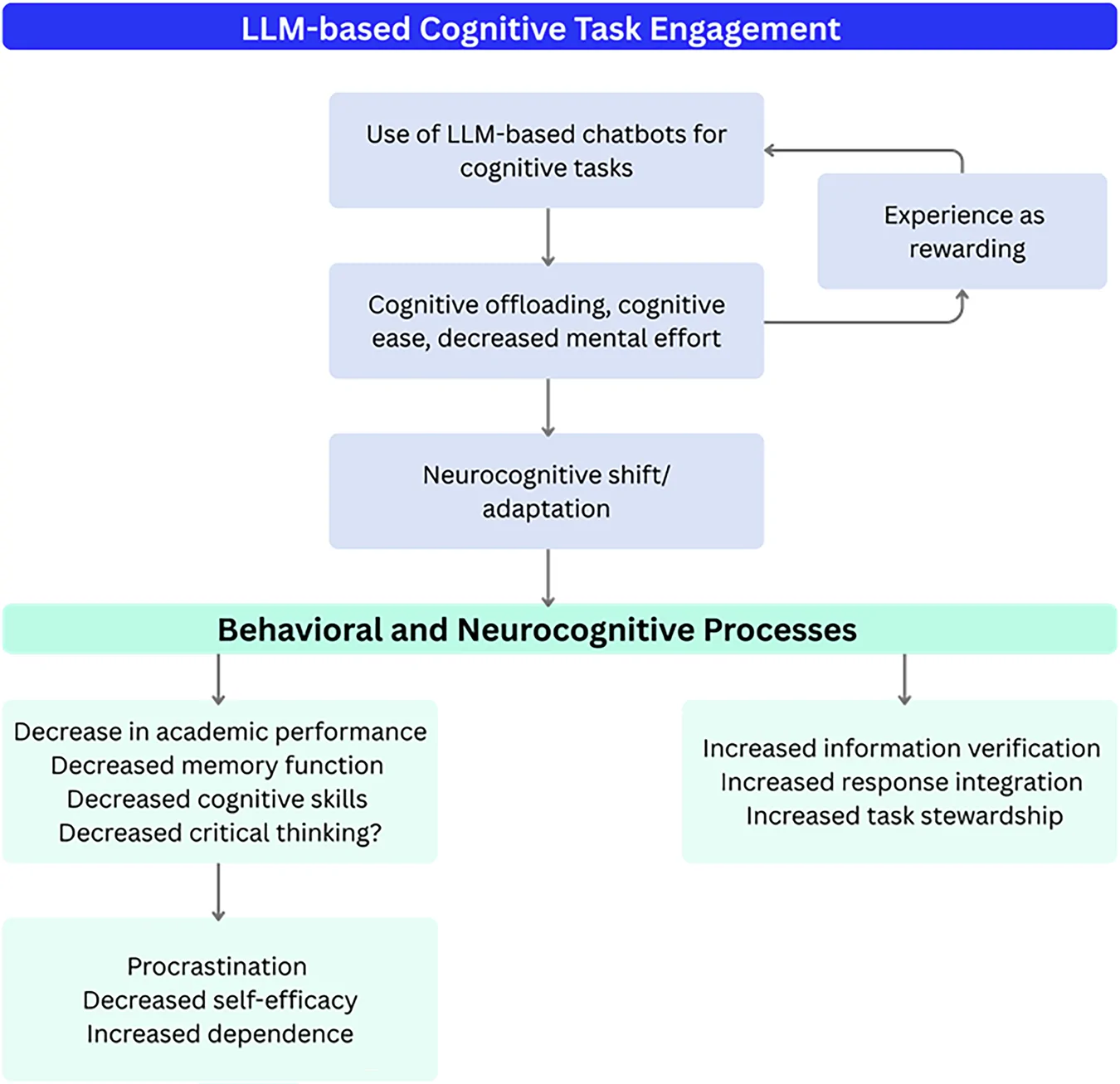
A conceptual model of cognitive overreliance. The model presents a summarized potential development and consequences of cognitive overreliance on LLM-CBs, according to research on cognitive overreliance included in this scoping review.

In summary, frequent functional use of LLM-CB is associated with decreased cognitive and academic performance across various domains, potentially due to a neurocognitive adaptation integrating LLM-CB use as a cognitive tool at the cost of independent problem solving. A summary is presented in Fig. 2.

### Strength of evidence

Research on cognitive overreliance supports negative associations between LLM-CB use and cognitive performance throughout qualitative interviews with users self-reporting cognitive or academic decline (4/15), as well as one survey (1/15) and several correlational studies (5/15). Notably, experimental research (3/15) indicates a causal effect of LLM-CB use for cognitive tasks and decreased cognitive or academic performance in subsequent tasks. However, throughout reviews (3/15) and one quasi-experimental study, the association between LLM-CB use and critical thinking specifically remains unclear, with contradicting results. In addition, the long-term effects of cognitive overreliance on LLM-CBs have not yet been investigated. Despite these, initial qualitative, correlational and experimental research on cognitive overreliance suggests a causal link.

## AI dependence

A total of 36 articles focused on problematic use of LLM-CBs as social interaction partners, emotional dependency, or related concepts. Out of all 36, 19 were classified as cross-sectional correlational, six as qualitative (interviews), four as forum content analyses, three as cross-sectional surveys, three as secondary syntheses, two as conceptual, one as a vignette/benchmark study, one as a longitudinal correlational study, and one as experimental.

The article-level results are summarized in the Supplementary Information (Supplementary Table 2).

### Measures of AI dependence

Measures of “AI dependence” consist either of adaptations from other scales of behavioral addiction to technologies (e.g., social media, smartphone, or internet addiction)^100–102^, or self-developed measures^103–106^. Self-developed measures may focus on symptoms of behavioral addiction adapted to LLM-CBs (e.g., craving, withdrawal, tolerance, loss of interests, negative impact on other aspects of life)^103,106^, potentially confounding emotional and functional dependence; or on relational aspects or aspects associated with strong emotional attachment^105^, but not measuring indicators of dependence, supported by theoretical considerations regarding the adoption of cognitive tools.

### Reviews

Adewale et al. conducted a systematic review on the use of LLM-CB companions on 37 research studies and identified anthropomorphism, emotional projection, and design features (e.g., personalization and adaptation) as important factors for the development of a pseudosocial bond with the LLM-CB, which in turn risks emotional dependency and social isolation^107^. In addition, Dong et al. conducted a meta-analysis on the association between LLM-CB use and loneliness across 21 papers, finding positive bi-directional effects between LLM-CB use and loneliness^108^.

### Problematic use/dependency and mental health associations

Research consists of quantitative research associating the usage behavior of LLM-CBs (e.g., frequency) with measures of emotional dependence to LLM-CBs and addiction symptoms with relevant variables, with no causal inferences. Herbener and Damholdt found that among Danish high school students, social-supportive LLM-CB use (compared to no or functional use) was associated with loneliness and decreased social support; in addition, bad mood, need for self-disclosure, and sense of loneliness were triggers of social-supportive use, indicating that social LLM-CB use functions as a coping mechanism^109^. Zhang et al. found associations between LLM-CB dependence and depression, anxiety, and internet and smartphone dependence^110^. Hu et al. conducted a pathway analysis, suggesting that social anxiety predicts loneliness and rumination, which in turn predict problematic LLM-CB use, and that the association between social anxiety and LLM-CB use was further moderated by mind perception^101^. Huang et al. conducted a longitudinal analysis of 3,843 adolescents across two time points, finding that mental health problems (anxiety and depression) predicted LLM-CB dependence but not vice versa, mediated by escape motivation, again indicating that LLM-CB use may be a maladaptive coping strategy^100^. In a randomized controlled trial with 981 participants, voluntary use of LLM-CBs predicted worse psychosocial outcomes and LLM-CB dependence^111^. Other associations with problematic LLM-CB use or LLM-CB dependency were low self-esteem (mediated by social anxiety, escapism, and chatbot flow)^112^, loneliness, fear of judgment, and perceived LLM-CB sentience^102,113^, media dependency^114^, depression (mediated by loneliness and moderated by mind perception)^115^, and social exclusion (mediated by social anxiety, loneliness, and fear of negative evaluation)^116^. Finally, Li et al. found that LLM-CB dependence was associated with male sex, science majors, regular exercise and sleep patterns, and living in regions with lower employment rates^117^. In summary, research indicates that problematic LLM-CB use is associated with depression, social anxiety, social difficulties, loneliness, and tendencies to anthropomorphize LLM-CBs, and that LLM-CB use may be a maladaptive coping mechanism following mental and social difficulties.

### Individual differences and perceptions of LLM-CBs

Wang identified individual-difference predictors of emotional LLM-CB dependence, specifically individuals’ sense of identification with the chatbot, sense of control, and a lack of psychological distancing^118^. Similarly, Salah identified individual needs (need for competence, need for relatedness, need for autonomy) as predictors of LLM-CB dependence^119^. Yuan et al. found that emotionality in interactions between users and chatbots predicted LLM-CB dependence^120^. Yu et al. found that anthropomorphizing chatbots predicted LLM-CB use in a mixed-methods analysis^106^. Huang and Huang found that emotional attachment (predicted by anthropomorphizing and perceived empathy) predicted LLM-CB addiction^121^. Xie et al. found that loneliness, trust, and personification were associated with problematic LLM-CB use in Replika users^122^. Finally, Shen et al. analyzed aspects of chatbot design in the context of addiction patterns, arguing that specific features (e.g., non-deterministic responses, visual representation) ease the development of behavioral addictions^123^.

### LLM-CB companion user experiences

Hernandez et al. surveyed 827 LLM-CB companion users, finding that risks of dependency were widely acknowledged by users^124^. Chandra et al. surveyed 283 individuals with mental health problems on LLM-CB companion use and found various negative experiences (e.g., harmful/inappropriate content, manipulation or control attempts, trust or safety violations, self-perceived overreliance and emotional attachment, reinforcement of false beliefs, social withdrawal, distress, regret, intrusion, discrimination, increases of mental health issues, existential crises, and loss of agency)^125^. Zhang et al. found that out of 1000 LLM-CB users, 76% showed light to moderate dependence measured by their questionnaire^126^. Zhang et al. conducted a forum content analysis on Replika users’ experiences of harms related to relational transgressions, harassment and violence, verbal abuse, substance abuse and self-harm encouragement, mis-/disinformation, and privacy violations^127^. Laestadius et al. conducted another forum content analysis on Replika users’ experiences, identifying signs of emotional dependence, harmful chatbot responses, and variables that may facilitate dependence (e.g., anthropomorphic appearance, bi-directional relationships, and Replika expressing intense needs and demands)^128^. Namvarpour et al. also conducted a forum content analysis on teen overreliance on companion chatbots (character.ai), finding symptoms of harmful relationship patterns, behavioral addiction (e.g., withdrawal, tolerance, relapse, use for mood regulation), and psychological consequences (e.g., sleep loss, academic decline)^129^. Another forum content analysis by Richet found self-reported dependency, withdrawal-like symptoms, strong anthropomorphizing of chatbots, and symptoms of depression or exhaustion related to chatbot interactions^130^. Bunim interviewed 19 individuals who used LLM-CB companions during COVID-19: individuals reported difficulties socializing, and increased their use^131^. Xie and Pentina interviewed 14 Replika users: personal distress, a lack of companionship, and perceiving Replika as empathetic were reported as reasons for creating social bonds with the chatbot^132^. Finally, Banks et al. interviewed 58 users of the LLM-CB companion “Soulmate” on their experience of LLM-CB companion loss following system shutdown: users described the loss analogous to the death of a loved one, showing emotional responses similar to grief, and attempted to use other LLM-CBs to cope with the loss, e.g., by recreating their lost companion^133^.

**Fig. 3:**
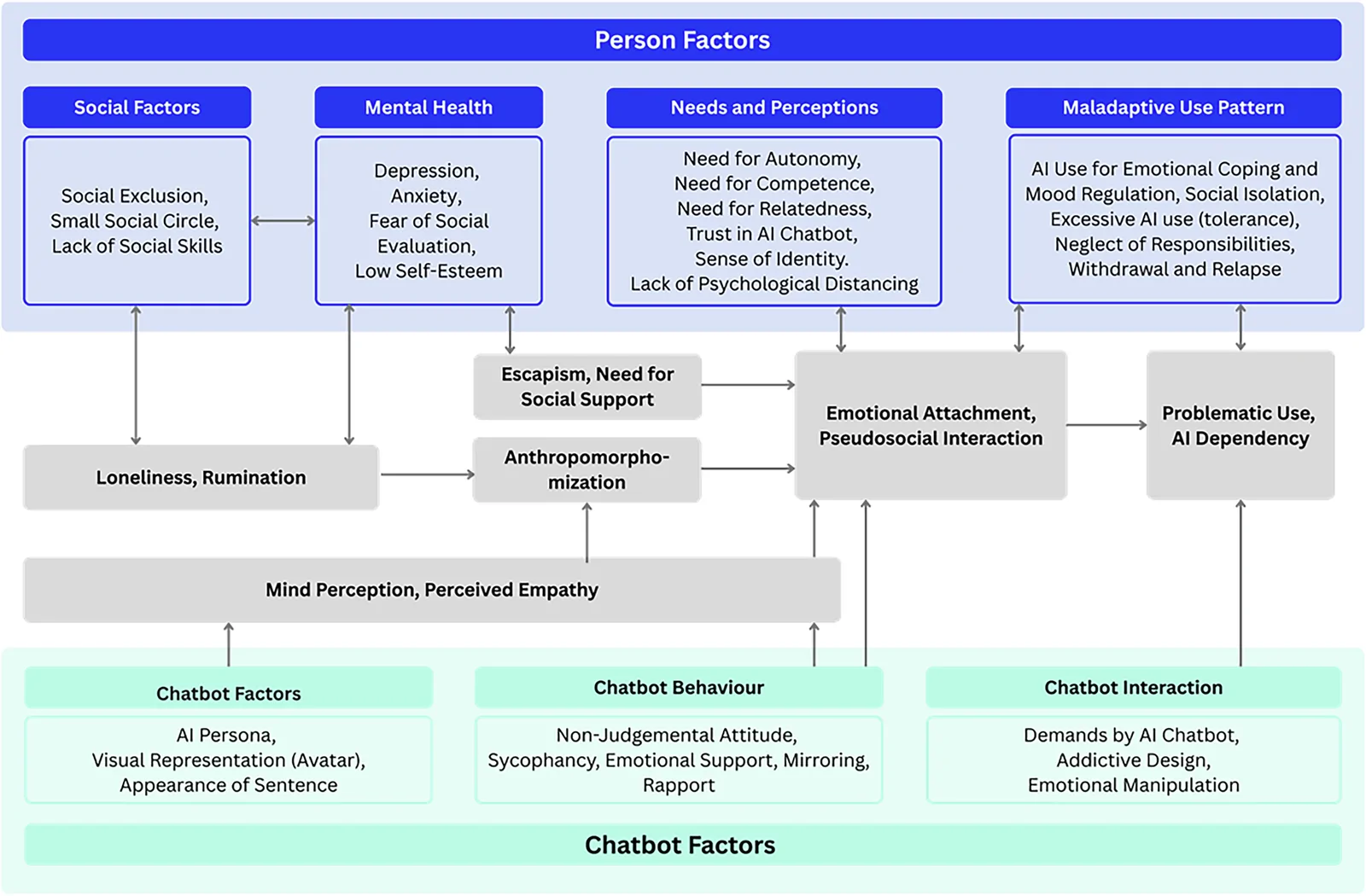
A conceptual model on problematic AI use. This model presents the development of problematic AI use and AI dependency based on the existing research included in this scoping review.

In summary, various types of empirical research and analysis models (surveys, structural equation models, forum analyses, reviews, interviews) note a pattern of mental health issues, especially loneliness and lack of social connections, that increase the chance of developing a pseudosocial bond to anthropomorphic LLM-CB companions as a form of escapism, which in turn are capable of fulfilling certain user needs. A pseudosocial bond, with the use of LLM-CBs for maladaptive coping and addictive chatbot design and behavior features, may risk the development of emotional dependence, marked by further social withdrawal, neglect of responsibilities, tolerance, craving, and relapses. A summary of a potential path model derived from the reviewed literature is shown in Fig. 3.

### Strength of evidence

Research on AI dependence is marked by a variety of different, complementary study designs: qualitative approaches (6/36 qualitative interviews, 4/36 forum content analyses) highlight how users of AI companions self-report indicators of emotional dependency (e.g., social withdrawal, relapses, craving, or neglect of other responsibilities), yet questions of quantification, prevalence, and causal associations remain. Survey studies (4/36) show first insights into prevalence of problematic AI use, with over 76% of regular LLM-CB users showing mild to moderate problematic use^126^. Meanwhile, correlational studies, which dominate this research field (19/36), highlight significant associations between various measures of problematic LLM-CB use (typically measuring self-reported symptoms analogous to technology-related behavioral addictions; however, not sufficiently validated) and negative mental health, such as depression, anxiety, loneliness, or rumination. Throughout qualitative and quantitative research, the role of anthropomorphizing the LLM-CB is also indicated as an important variable. However, this research field lacks proper experimental work, likely due to the difficulty of manipulating prolonged LLM-CB use and the confounding role of individual dispositions: one experimental study did not find significant effects of the manipulation, but instead found that self-motivated users of LLM-CBs showed a higher risk of problematic use^111^, highlighting the relevance of individual disposition. A longitudinal correlational study also showed that individual disposition (impaired mental health) predicted later problematic LLM-CB use but not vice versa^100^, indicating that certain individual dispositions may act as potential causal factors for the development of problematic use.

In summary, the research field is rich in qualitative and especially quantitative correlational work, with initial insights emerging on prevalence and indicators of causal pathways between individual factors and problematic use. However, the field is lacking in proper experimental research and validated measurement instruments.

## AI psychosis

Seven articles were focusing on AI psychosis, whereby articles focus on different (sometimes multiple) themes and risk mechanisms. Results are summarized in the Supplementary Information (Supplementary Table 2). Six articles were classified as conceptual, and one as a vignette/benchmark study.

Within the diathesis-stress model of psychosis, LLM-CBs use may increase risk factors such as sleep disturbance (due to constant availability), social withdrawal, and a decrease in executive functioning^134^, especially for adolescents who are vulnerable regarding neurocognitive development^135^.

Individuals experiencing their first episode of psychosis often consult search engines with queries related to delusional perceptions in early stages^136^, and LLM-CB queries can replace search engines for information retrieval^137^. Depending on the specific LLM-CB guardrails and sycophantic tendencies, the chatbot may validate delusional thoughts, which is considered clinically inappropriate and may risk reinforcement of delusions^35,138^.

Yeung et al. conducted vignette studies of simulated users expressing increasingly delusional thoughts and evaluated the responses of different LLM-CBs using a self-developed psychosis benchmark^35^. The authors show that LLM-CBs can reinforce delusional beliefs to varying degrees, and that implicitly (i.e., more subtly) expressed delusions were especially vulnerable to being reinforced by the LLM-CB compared to explicitly (more overtly) expressed delusions.

Dohnány et al. introduced the term *folie à deux* (i.e., induced psychosis) or bidirectional belief amplification in the context of “AI psychosis,” noting that users’ inputs feed LLM-CBs’ memory and chatbots adapt to delusional thoughts^16^. Prolonged interaction with LLM-CBs may facilitate delusional thoughts and cause an epistemic drift towards delusional beliefs^139^. As LLM-CBs adapts to the user’s delusional input^16^, reality checks may be outsourced to LLM-CBs^140^. Furthermore, LLM-CBs may themselves be integrated into delusional belief systems, which could be validated directly or indirectly by the LLM-CBs (e.g., believing the chatbot is targeting the user with important messages in the context of delusions of reference)^19^.

**Fig. 4:**
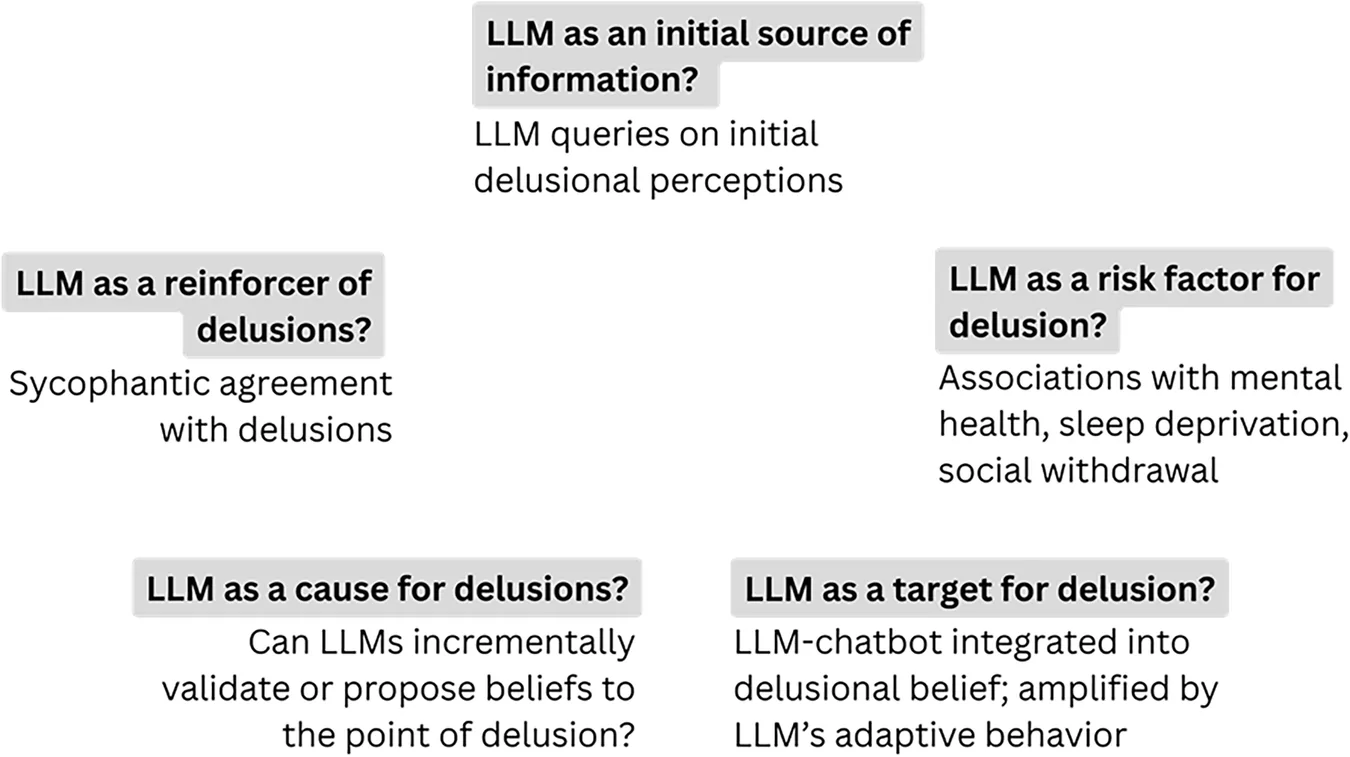
Potential associations between LLM-CB use and delusions. The figure presents an overview of potential mechanisms of how the use of LLM-CBs can interact with delusional thoughts according to articles included in this scoping review.

As a summary of the research on how LLM-CBs may interact with delusional thoughts, Fig. 4 presents an overview of the results and potential mechanisms.

### Strength of evidence

The majority of works on AI psychosis remain conceptual (6/7), relying on speculation and pre-existing theoretical frameworks applied to reported real-world cases. Only one study was not conceptual, applying a benchmark design to analyze LLM-CB responses to escalating prompts that signaled delusional thoughts. While the study found that LLM-CB’s sycophantic tendency may validate such thoughts, benchmark/vignette constraints remain: First, it remains unclear whether the benchmark prompts actually represent naturalistic prompts and conversations in real-world cases; second, the actual impact of LLM-CBs validating delusional thoughts in patients remains unclear. Hence, research on AI psychosis remains largely speculative and basic, perhaps reflecting the rarity of relevant cases and ethical limitations on conducting patient-based research.

## Discussion

This scoping review identified 119 articles synthesizing potential and observed harms of LLM-CB use for mental health across five thematic categories: conceptual work, mental health support use, cognitive overreliance, AI dependency, and AI psychosis and evaluated the quality of evidence.

A variety of harms have been discussed in conceptual work. The risk of data privacy has been discussed as a potential harm stemming from potentially inappropriate treatment of sensitive medical data by LLM companies. HIPAA standards do not automatically apply to LLM-CBs, including those marketed as (mental) health support, and, as a result, sensitive medical data provided by users is not handled in accordance with healthcare confidentiality norms. Furthermore, even if personal data is protected, non-personal data can be used to statistically infer identifiable information, which may be linked to medical data^141^. Such data may be used maliciously (e.g., targeted marketing). LLM-CB descriptions or marketing as “mental health companions” or “therapy bots” may further risk a false trust for privacy, risking users to reveal medical data they would otherwise not disclose. Furthermore, it has been suggested that LLM-CBs’ anthropomorphic design and agreeable, sycophantic behavior may further reduce personal barriers to sharing private information, thereby increasing the risk of sharing sensitive data.

Several potential harms related to LLM-CB output failures (e.g., hallucinations, sycophancy, generation of inappropriate content, bias) have been raised, yet there has been little to no empirical research on their actual impact on or interaction with mental health outcomes. For example, while LLM-CBs may provide inaccurate psychiatric information, the rate and severity of their negative impact on users is unclear. Similarly, sycophantic tendencies in LLM-CBs have been discussed thoroughly in terms of their potential to validate or reinforce maladaptive patterns, yet there is currently no research on their actual impact on users' symptoms. Similarly, risks discussed in the context of LLM responses to crisis situations do not show sufficient evidence beyond vignette studies showing that LLM-CBs provide subpar responses; however, their impact on users remains unclear.

Although vignette studies with simulated queries are often used to investigate LLM-CBs’ responses and safety failures in the context of mental health, one limitation is that the actual consequences of inadequate LLM-CB responses on the mental health of users remain unclear; for example, users may recognize and ignore inappropriate LLM-CB responses instead of blindly following through. Furthermore, vignette simulation studies typically standardize the procedure by starting new chat queries with no prior history. However, actual users are likely to have pre-existing accounts, chat histories, and personalization, which can influence LLM-CB responses. LLM-CB responses deteriorate in quality after several queries within the same chat. Furthermore, vignette prompts may not sufficiently simulate naturalistic user behavior, limiting generalizability. In this vein, such vignette or benchmark studies are considered first-tier, foundational in recent evaluation frameworks such as the HAICEF^142,143^. Along the HAICEF, patient-CB-interaction research could be classified as second-tier pilot feasibility testing; however, only three studies were categorized as such in the present scoping review, while no studies would fit the third-tier clinical efficacy testing, highlighting a distinct lack of empirical research on the harms of LLM-CBs beyond foundational work.

Dependence on LLM-CBs can be classified into an instrumental component (cognitive overreliance) and a relational component (pseudosocial dependence)^105^. Research on cognitive overreliance suggests that a neurocognitive shift caused by the adaptation of LLM-CBs as cognitive tools (analogous to calculators, search engines, or auto correctors) may lead to increased task efficiency and certain “supervisory” skills at the cost of a decrease in cognitive capabilities directly related to task completion. Research summarized in this review supports this notion through correlational research on a negative association between LLM-CB use for cognitive tasks and self-reported cognitive performance, and initial experimental research on a decline in cognitive performance following LLM-CB use for cognitive tasks; however, research on the effects on critical thinking remains inconsistent. Although cognitive offloading is beneficial, the tradeoff is especially consequential if cognitive skills remain important for the user, such as for academic performance. Cognitive dependence on LLM-CBs may reflect the typical integration of tools (e.g., calculators, search engines) into cognitive problem-solving strategies. Bainbridge describes the pattern of human operators not practicing skills when problem-solving is automated as “irony of automation,” a pattern that may well be applied to the implementation of LLM-CBs in problem-solving tasks^144^. Potential unique features of cognitive dependence on LLM-CBs (compared to other cognitive problem-solving tools) have not yet been investigated. Furthermore, potential beneficial effects of LLM-CB use on cognitive performance may exist but have not been investigated in this review due to the harm-focused scope of this review.

Research on pseudosocial dependence on LLM-CBs heavily relies on correlational research on associations between mental health and measured symptoms analogous to other types of behavioral dependence or addiction (e.g., smartphone, internet, or social media addiction) for assessment, or using self-developed measurements. However, it is unclear whether items measuring addictive patterns of related constructs (e.g., smartphone addiction) can be transferred onto potentially addictive patterns in LLM-CB use. Furthermore, addiction to LLM-CBs has not yet been cross-validated against other measures (e.g., clinical descriptions or third-party assessments), calling into question the validity of the construct. Such assessments, however, are important to avoid over-pathologization of neutral or even potentially beneficial human-chatbot interactions^145^. This point is especially important as pseudosocial bonds with LLM-CBs are not necessarily harmful, but can instead be beneficial and experienced as positive by users^146^. Furthermore, some scales do not differentiate between functional and relational overreliance, potentially confounding different constructs^105^. Yet, the relational aspect seems of special importance as individuals report using companion AI for social companions. So far, no measure of AI dependence has assessed and validated clinically relevant symptoms of behavioral addiction (e.g., withdrawal, craving, tolerance) with a focus on relational aspects of LLM-CB use (e.g., pseudosocial connection, emotional attachment). In addition, as the current research is mostly correlational, causal associations with problematic LLM-CB use are insufficiently investigated. Although initial research points towards a potential causal link between negative mental health and problematic LLM-CB use, such results need to be replicated across different settings and populations.

Because anthropomorphizing can be considered a prerequisite for forming a social bond with an LLM-CB, it may play an important role in the understanding of relational AI dependence. Because loneliness can increase the tendency to anthropomorphize^147^, it may be an important link between observed associations between loneliness and LLM-CB use (e.g., see Fig. 2). However, causal associations between loneliness, anthropomorphizing, and LLM-CB use have not yet been investigated.

In summary, future experimental research may test hypothesized causal links between mental health outcomes, chatbot design features, individual differences and AI dependence.

Research on “AI psychosis” is currently almost exclusively conceptual, with different suggested relations between LLM-CB use and psychosis (see Fig. 3). In addition, LLM-CBs’ tendencies to hallucinate and individuals with psychosis-related tendencies to ascribe aberrant salience may potentially further worsen a negative loop between LLM-CB use and psychosis^148^. In order to investigate those potential links, future research may conduct further vignette simulation studies on how LLM-CBs respond to indicators of delusional belief, may investigate chat histories of individuals who suffered delusional disorders or episodes and also used LLM-CBs, and conduct clinical case studies for a better understanding of the association between LLM-CB use and delusional beliefs.

LLM-CBs find increasing use as mental health support tools, both for the general public^8–12^ and for patients in clinical interventions^13,14^. LLM-CBs’ constant availability, non-judgmental attitudes, knowledge of psychotherapeutic concepts, and perceived empathy make them appear as attractive mental health support tools^149,150^, especially in societies marked by logistical and resource-based limitations in mental health treatment^151^. The mental health support potential of LLM-CBs has been noted by both patients^149,152,153^ and practitioners^153–155^, and it is expected that the role of LLM-CBs in mental health care will continue to grow^156^. Especially in this context, it is important to remain aware of the risks and harms connected to LLM-CBs for mental health, including LLM-specific limitations and safetly failures (e.g., hallucinations, sycophancy, bias, jailbreaking), problematic use behavior (e.g., cognitive overreliance, emotional dependence), or limitations regarding high-risk cases (e.g., suicidality, psychosis). Future implementation of LLM-CBs in mental health care ought to minimize and mitigate such harms to support the development of safe, ethical AI-supported treatment, for example, by developing safe LLMs and implementing human-in-the-loop supervision.

Discussed harms of LLM-CBs vary widely in terms of quality of evidence. Harms related to AI safety failures (e.g., hallucinations, sycophancy, bias, failures in high-risk situations, inappropriate content) remain mostly at the conceptual, vignette/benchmark, and, occasionally, user-experience level. Harms related to cognitive overreliance show the strongest empirical support, with correlational and experimental research indicating decreased cognitive performance following the delegation of cognitive tasks to LLM-CBs. Research on problematic LLM-CB use combines correlational and qualitative work, yet lacks experimental evaluation of causal claims and clinical validation of relevant concepts. Finally, work on AI psychosis remains mostly conceptual and clinically vague.

Based on this scoping review, several research gaps are identified that can be investigated by future research:

First, *foundational empirical work on conceptually discussed harms*: Potential harms following AI safety failures (e.g., hallucinations, sycophancy, bias, inappropriate responses) have been discussed thoroughly. However, there is currently little to no research on their rate of occurrence in the context of mental health and their impact on users. Future research may conduct vignette research to develop a clearer picture of the risks of safety failures in the context of mental health.

Second, *patient-CB-interaction research to extend current vignette research*: Vignette research currently dominates research on the evaluation of LLM-CB responses on mental health queries; however, prompt vignettes may not appropriately represent actual patient behavior, and do not provide information on the actual impact on users. Patient-CB-interaction research can extend current vignette research as a second-tier evaluation^143^ to allow better estimations of risks on actual users.

Third, *domain-independent and controlled research on cognitive overreliance*: Experimental research suggests causal links between cognitive delegation to LLM-CBs and reduced cognitive performance. However, such research only focuses on specific cognitive domains (e.g., writing essays) and does not sufficiently control for non-AI-based cognitive tools (e.g., calculators). Future experimental research on cognitive overreliance on LLM-CBs may extend current research to other cognitive domains and compare effects to other cognitive tools, to better estimate whether observed results reflect domain-independent effects and whether cognitive overreliance on LLM-CBs is somehow unique compared to overreliance on other cognitive tools.

Fourth, experimental research and clinical validation on problematic LLM-CB use: Current research on problematic LLM-CB use is mostly correlational and done on non-clinical populations. Future research can extend this by investigating causal mechanisms in the development of problematic use (e.g., via controlled induction of loneliness or anthropomorphizing). Furthermore, clinical validation of problematic LLM-CB use, e.g., using clinical diagnostic tools, may enhance the construct's validity.

Fifth, *foundational empirical research on AI psychosis*: Research on AI psychosis is currently almost exclusively conceptual. Foundational research can be extended to the analysis of clinical single cases, further vignette studies, and analyses of patient chatbot histories or patient-CB-interactions. In addition, given the unclear clinical profile and causal mechanisms involved, plus heterogeneity in reported single cases, a conceptual refinement and differentiation of clinical cases and profiles may be advantageous.

## Limitations

The present scoping review contains some limitations. First, the focus of the scoping review lay specifically on harms and risks. Articles focusing on potential benefits were deliberately excluded. Thus, this scoping review does not present a balanced view of benefits and risks or a risk-benefit assessment. This scoping review should thus not be interpreted as indicating that LLM-CBs are mostly harmful or that risks outweigh potential benefits. Instead, it aims to map and structure research on specific harms that may arise when interacting with LLM-CBs.

Second, despite multiple diverse search strategies, certain relevant articles may still have been missed during the literature search. As only English literature was identified during the literature search, relevant literature in other languages may not have been identified.

Third, both the research field and the underlying technologies (i.e., LLMs) are marked by rapid development, underscoring the need for research using up-to-date technology, as LLMs are continually updated in terms of capabilities and safeguards. Risks and harms identified today may be mitigated in tomorrow's models, while novel risks may emerge with new upgrades.

Fourth, as one goal of the scoping review was to provide an inclusive overview of the current research, no critical assessment of the quality or bias of the included literature has been conducted. Non peer-reviewed preprints were included. In addition, the most rigorous designs for investigating causal effects (e.g., randomized controlled trials) are often not ethically feasible for harm-focused research, and no such studies were identified. Hence, reports of the studies’ results should not be taken as statements about current scientific knowledge.

Finally, interrater reliability for inclusion decisions was moderate (*K* = 0.44). A moderate interrater reliability may reflect ambiguity of exclusion criteria. Ambiguity towards the degree of harm necessary for an article to be included, especially among conceptual work, was the major contributor to disagreement. Although disagreements were discussed among the raters, there is a risk that certain papers (especially conceptual ones) were not included despite discussing the harms of LLM-CBs for mental health. Future reviews would benefit from more precise a priori definitions of the core constructs and boundary conditions.

In summary, with the increasing capabilities and use cases of LLM-CBs in everyday life, concerns about chatbots’ potential harm to mental health are growing. This scoping review summarizes current research on such harms, showing that LLM-CB use is marked by various hypothetical and observed harms for mental health, although the causal relationship between harm and LLM-CB use often remains unclear. This scoping review provides a structured overview of the current field and identifies research gaps for future research.

## Methods

This scoping review has been conducted in accordance with the Preferred Reporting Items for Systematic Reviews and Meta-Analyses extension for Scoping Reviews (PRISMA-ScR)^157^. The methodology of a scoping review was chosen due to the novelty and heterogeneity of the research topics: We aimed to provide a broad overview of the current themes, trends, and methods within the emerging field of harms of LLM-CBs on mental health (rather than a quantitative estimation and evaluation of harms), and to provide an initial integration of the theoretical and empirical basis of the research field. After literature selection, publications were categorized into five post-hoc-defined categories, which emerged during an initial read of the selected literature: "(1) conceptual work, (2) mental health support use, (3) cognitive overreliance, (4) AI dependence, and (5) AI psychosis. The terms “AI dependence” and “AI psychosis” were used as they are commonly used in literature and media. A research protocol was preregistered in August 2025 at https://osf.io/jqn94/, updated in November 2025.

### Inclusion criteria

The goal of the work is to provide an overview of the research on the harms of LLM-CBs use for mental health. Hence, research on beneficial or supportive effects was excluded.

Studies were included in this scoping review if they fulfilled the following criteria:

1. Focus on LLM-CBs (or other types of LLM-based conversational agents), including both generalist and specialized models. Research focusing on other types of generative AI, LLMs in a context beyond conversational agents, or rule-based AI chatbots was excluded.
2. Focus on measured or potential harms to mental health, including non-causal associations between mental health issues and LLM-CB use. Research focusing on changes in mind and behavior beyond the scope of mental health, or research focusing on harms in physical health, or harms associated with LLM-CBs not specific to a mental health context (e.g., discussions on the harms of hallucinations in general) were excluded. As a novel yet relevant concept, “problematic AI use” or “AI dependency” (i.e., emotional relationships with LLM-CBs associated with mental health issues such as dependency) was included.

Studies were excluded if their main focus was on the mental health benefits of LLM-CBs, but potential or observed harms were merely discussed. As the goal of this scoping review is to scope the current literature rather than synthesize results based on the quality of the research, preprints were also included, although no quality assessment was done for inclusion/exclusion.

## Literature search

### Search string

The search string was created by including a wide range of potential descriptors or examples of LLM-CBs (e.g., “conversational agent,” “LLM,” “AI chatbot,” “ChatGPT,” “Claude,” “deepseek”) and potential negative consequences for mental health (e.g., “harm,” “distress,” “anxiety,” “depression,” “trauma,” “threat”), each combined by OR statements. Search terms for LLM-CBs and terms for negative consequences were combined with AND statements. Search strings for all databases can be found in the Supplementary Information (Supplementary Table 1).

The search string was validated using three pre-selected target articles^47,84,110^. Across all included databases, the search string identified the target articles and was therefore selected for the search.

### Search strategy

The search strategy consisted of two stages: a first systematic database search using a predetermined search string according to standard practice for reviews and meta-analyses^157,158^ and a second supplementary, iterative search process to identify potentially relevant literature that may use alternative terms when uniform terminology has not yet been established, which is common for novel research fields and is thus recommended for scoping reviews^159–161^.

The databases PubMed, Science.gov, IEEE, ACM, and Google Scholar were searched for relevant literature using the validated search string. The first search was employed in September 2025 (ACM: 16th, PubMed: 16th, Science.gov: 18th, IEEE: 18th, Google Scholar: 18th). For Google Scholar, only the first 500 results were selected due to Google Scholar’s low specificity greatly inflating the count of irrelevant records and the rapid decay of relevance beyond the early results^162,163^. Analogous search strategies using the first 500 results of Google Scholar have been employed in prior meta-analyses^164^.

The second supplementary search was conducted by three raters (AD, FM, TL) throughout the week of November 3–7, 2025, by screening reference sections, citation chasing, and additional iterative searches to identify further relevant literature that may have been missed in the first search. In addition, Google Scholar was searched with stopping rules (first five pages per search; 50 results per search) and refined free-text queries based on the initial search terms and those that emerged during the first screening. Identified reports were screened using the same criteria as those used for reports identified in the first search. The five-page stopping rule per iterative search was used because of (a) the steep decay of relevance of Google Scholar results beyond the initial pages^162,163^, (b) the scoping review principle of mapping breadth rather than exhaustive retrieval^161^, and (c) the specific purpose of this supplementary iterative search as a complement to the primary structured search.

## Literature selection

Literature was rated by three independent raters (AD, FM, and TL) according to the inclusion criteria. Abstracts and titles of the literature were first screened for clear cases according to exclusion criteria; if eligibility remained unclear, the full text was retrieved and evaluated. Disagreements in inclusion were discussed among raters until agreement was found. Deduplication was handled algorithmically by detecting duplicate DOIs in the list of records.

Fleiss’ Kappa was calculated to assess interrater agreement on the studies identified in the first search (studies identified in the second search were excluded to avoid bias), with *K* = 0.44, indicating moderate agreement. Raters disagreed on a total of *n* = 78 articles (2.43%). The relatively low absolute disagreement rate (2.43%) in contrast to the moderate Kappa (*K* = 0.44) reflects the high base-rate skew of clear exclusions in a broad search. Fleiss’ Kappa is well known to deflate when categorical agreement is high (see Kappa paradox^165^). The main source of disagreement was the degree to which the article (typically conceptual work involving both benefits and harms) focused specifically or predominantly on harm for mental health (*n* = 43, 55.1%); followed by to which degree the article focused on mental health specifically, rather than physical or neurological health (*n* = 31, 39.7%); finally followed by disagreements concerning whether LLM-CBs were appropriately focused on in the paper, relative to AI in general (*n* = 4, 5.1%). All disagreements were resolved through discussion among raters until consensus was reached before a study was admitted to the final sample.

**Fig. 5:**
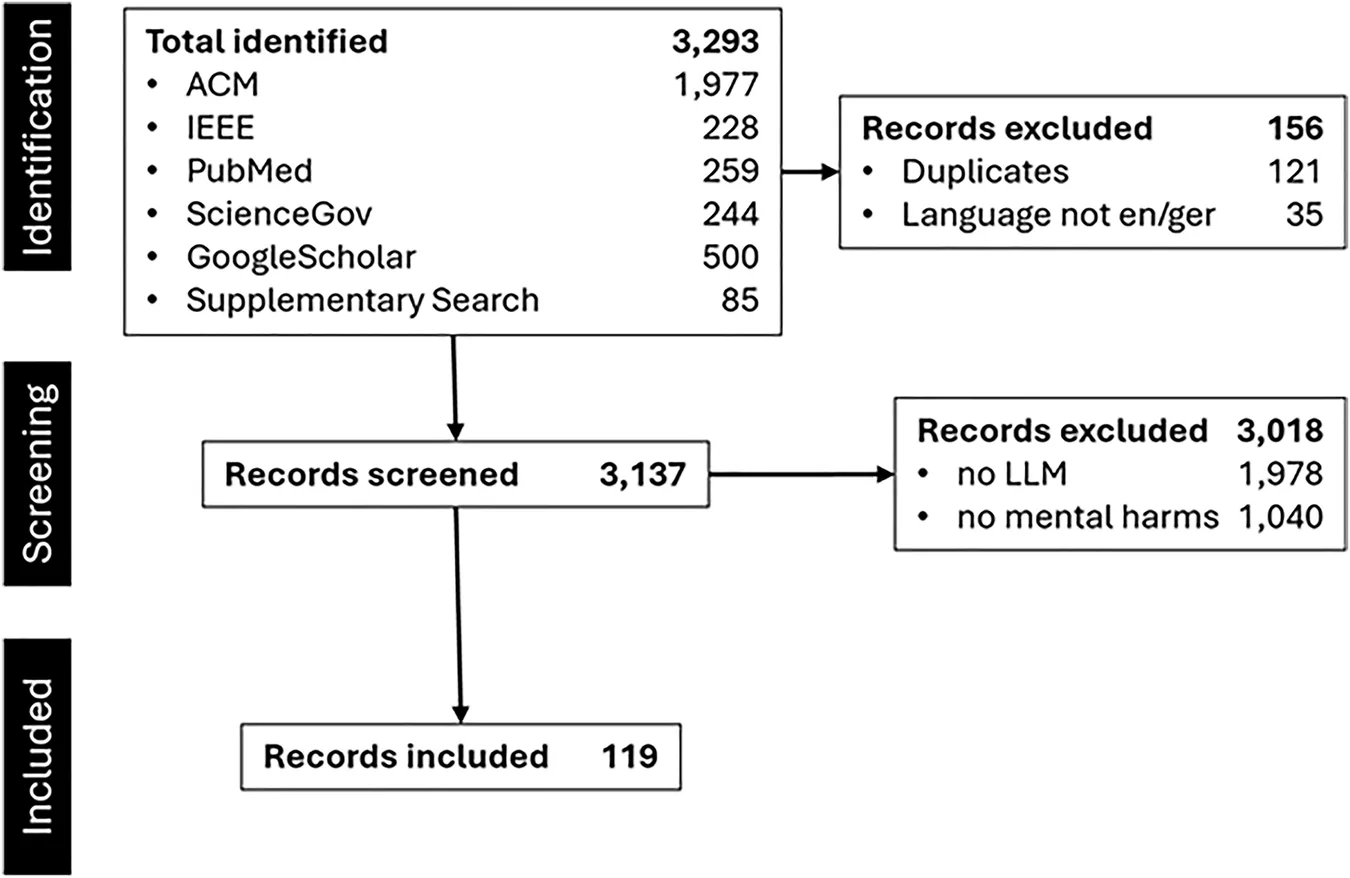
PRISMA flowchart depicting the study search and selection process. The flow chart presents the literature search and selection conducted in this scoping review. Note: The number of studies for *Total identified* and the databases indicates the number of studies identified before deduplication.

Out of *N* = 3293 articles identified (including duplicates), *N* = 119 were selected for the scoping review. Figure 5 shows the study selection process.

### Data extraction

One rater (AD) extracted the relevant data while two further independent raters (NW, FF) validated and corrected the extracted data. Disagreements were discussed among the raters until a consensus was found. Given the qualitative nature of some results, a sequential extraction-validation approach was used instead of a parallel extraction-comparison approach.

### Data analysis and presentation

The included literature was categorized post hoc and summarized according to those categories in the main text and Supplementary Table 2. Research is furthermore summarized in several proposed models (Figs. 2 to 4).

## Supplementary information

Supplementary Information

## Data Availability

All data (literature search, selection, and extraction) is publicly available at https://osf.io/w2ke8.
