# Publisher Correction: A scoping review on the mental health harms of LLM-based chatbots

**DOI:** 10.1038/s41746-026-03240-x

**Published:** 2026-09-14

**Authors:** Alexander Diel, John Torous, Pim Cuijpers, Jens Kleesiek, Felix Nensa, Niels Weber, Franziska Faust, Tania Josan Lalgi, Finley Sam Mellis, Martin Teufel, Alexander Bäuerle

**Affiliations:** 1 Clinic for Psychosomatic Medicine and Psychotherapy, LVR-University Hospital Essen, University of Duisburg-Essen, Essen, Germany; 2 Center for Translational Neuro- and Behavioral Sciences, University of Duisburg-Essen, Essen, Germany; 3 Beth Israel Deaconess Medical Center, Harvard Medical School, Boston, MA USA; 4 Department of Clinical, Neuro and Developmental Psychology, Amsterdam Public Health Research Institute, Vrije Universiteit Amsterdam, Amsterdam, The Netherlands; 5 Babeș-Bolyai University, International Institute for Psychotherapy, Cluj-Napoca, Romania; 6 Institute for AI in Medicine (IKIM), University Hospital Essen, Essen, Germany

**Keywords:** Diseases, Health care, Psychology, Psychology

Correction to: *npj Digital Medicine* https://doi.org/10.1038/s41746-026-03054-x, published online 20 August 2026

In this article, the order of Figs. 1–4 was inadvertently interchanged. The original article has been corrected.

