# Supplementary material for: A scoping review on the mental health harms of LLM-based chatbots
Source: NPJ Digit Med. 2026 Aug 20;9:644. doi: 10.1038/s41746-026-03054-x (PMC13493897; doi:10.1038/s41746-026-03054-x)
Supplement: Supplementary file 1 [file 41746_2026_3054_MOESM1_ESM.pdf]

Supplementary information

Supplementary Table 1. Search string used for the literature search.

PubMed, IEEE, Science.gov, Google Scholar	
Terms related to artificial intelligence systems	"ai" OR "llm" OR "artificial intelligence" OR "genAI" OR "large language model" OR "gpt" OR "palm" OR "chatgpt" OR "llama" OR "claude" OR "gemini" OR "grok" OR "deepseek" OR "mistral" OR "NLP" OR "natural language processing" OR "human-ai" OR "ai-human" OR "Replika" AND
Terms related to conversational agents	"chat*" OR "conversational agent" OR "companion" OR "chat" OR "bot" OR "bots" OR "conversational agents" OR "companions" AND
Terms related to negative effects	"harm" OR "harm*" OR "threat" OR "threat*" OR "misus*" OR "disus*" OR "manipula*" OR "parasocial" OR "parasocial*" OR "violen*" OR "negative impact" OR "negative effect" OR "illusion*" AND
Terms related to mental health	"anxiety" OR "anxious" OR "depressed" OR "depression" OR "depressive" OR "depressing" OR "distress" OR "distressed" OR "distressing" OR "isolating" OR "isolation" OR "isolated" OR "lonely" OR "loneliness" OR "concern" OR "concerned" OR "worry" OR "worrying" OR "ruminating" OR "rumination" OR "insomnia" OR "insomnic" OR "psychosis" OR "psychotic" OR "disorder" OR "disordered" OR "trauma" OR "traumas" OR "traumatizing" OR "traumatising" OR "traumatized" OR "traumatised" OR

	"traumatic" OR "suicide" OR "suicides" OR "suicidal" OR "victim" OR "victimized" OR "victimizing" OR "mental health" OR "emotional state" OR "psychiatry" OR "psychology" OR "panic" OR "panics" OR "panicked" OR "anorexia" OR "anorexic" OR "bulimia" OR "bulimic" OR "binge eating" OR "harassed" OR "harassing" OR "harassment" OR "harass" OR "stress" OR "stressed" OR "stressing" OR "stressful" OR "stressfully" OR "mental health" OR "well-being" OR "well being" OR "eating disorder" OR "eating disorders" OR "psychosomatic" OR "somatization" OR "hypochondria" OR "emotional regulation" OR "affective regulation" OR "abuse" OR "abusive" OR "abused" OR "abusing" OR "emotion regulation" OR "affect regulation"
Arxiv	
Terms related to artificial intelligence systems	"ai" OR ALL:"llm" OR ALL:"artificial intelligence" OR ALL:"genAI" OR ALL:"large language model" OR ALL:"gpt" OR ALL:"palm" OR ALL:"chatgpt" OR ALL:"llama" OR ALL:"claude" OR ALL:"gemini" OR ALL:"grok" OR ALL:"deepseek" OR ALL:"mistral" OR ALL:"NLP" OR ALL:"natural language processing" OR ALL:"human-ai" OR ALL:"ai-human" OR ALL:"Replika"
Terms related to conversational agents	"chat*" OR ALL:"conversational agent" OR ALL:"companion" OR ALL:"chat" OR ALL:"bot" OR ALL:"bots" OR ALL:"conversational agents" OR ALL:"companions"

Terms related to negative effects	"harm" OR ALL:"harm*" OR ALL:"threat" OR ALL:"threat*" OR ALL:"misus*" OR ALL:"disus*" OR ALL:"manipula*" OR ALL:"parasocial" OR ALL:"parasocial*" OR ALL:"violen*" OR ALL:"negative impact" OR ALL:"negative effect" OR ALL:"illusion*?"
Terms related to mental health	"anxiety" OR ALL:"anxious" OR ALL:"depressed" OR ALL:"depression" OR ALL:"depressive" OR ALL:"depressing" OR ALL:"distress" OR ALL:"distressed" OR ALL:"distressing" OR ALL:"isolating" OR ALL:"isolation" OR ALL:"isolated" OR ALL:"lonely" OR ALL:"loneliness" OR ALL:"concern" OR ALL:"concerned" OR ALL:"worry" OR ALL:"worrying" OR ALL:"ruminating" OR ALL:"rumination" OR ALL:"insomnia" OR ALL:"insomnic" OR ALL:"psychosis" OR ALL:"psychotic" OR ALL:"disorder" OR ALL:"disordered" OR ALL:"trauma" OR ALL:"traumas" OR ALL:"traumatizing" OR ALL:"traumatising" OR ALL:"traumatized" OR ALL:"traumatised" OR ALL:"traumatic" OR ALL:"suicide" OR ALL:"suicides" OR ALL:"suicidal" OR ALL:"victim" OR ALL:"victimized" OR ALL:"victimizing" OR ALL:"mental health" OR ALL:"emotional state" OR ALL:"psychiatry" OR ALL:"psychology" OR ALL:"panic" OR ALL:"panics" OR ALL:"panicked" OR ALL:"anorexia" OR ALL:"anorexic" OR ALL:"bulimia" OR ALL:"bulimic" OR ALL:"binge eating" OR

	ALL:"harassed" OR ALL:"harassing" OR ALL:"harassment" OR ALL:"harass" OR ALL:"stress" OR ALL:"stressed" OR ALL:"stressing" OR ALL:"stressful" OR ALL:"stressfully" OR ALL:"mental health" OR ALL:"well-being" OR ALL:"well being" OR ALL:"eating disorder" OR ALL:"eating disorders" OR ALL:"psychosomatic" OR ALL:"somatization" OR ALL:"hypochondria" OR ALL:"emotional regulation" OR ALL:"affective regulation" OR ALL:"abuse" OR ALL:"abusive" OR ALL:"abused" OR ALL:"abusing" OR ALL:"emotion regulation" OR ALL:"affect regulation"

Supplementary Figure 1. Decision tree used for the categorization of the articles.

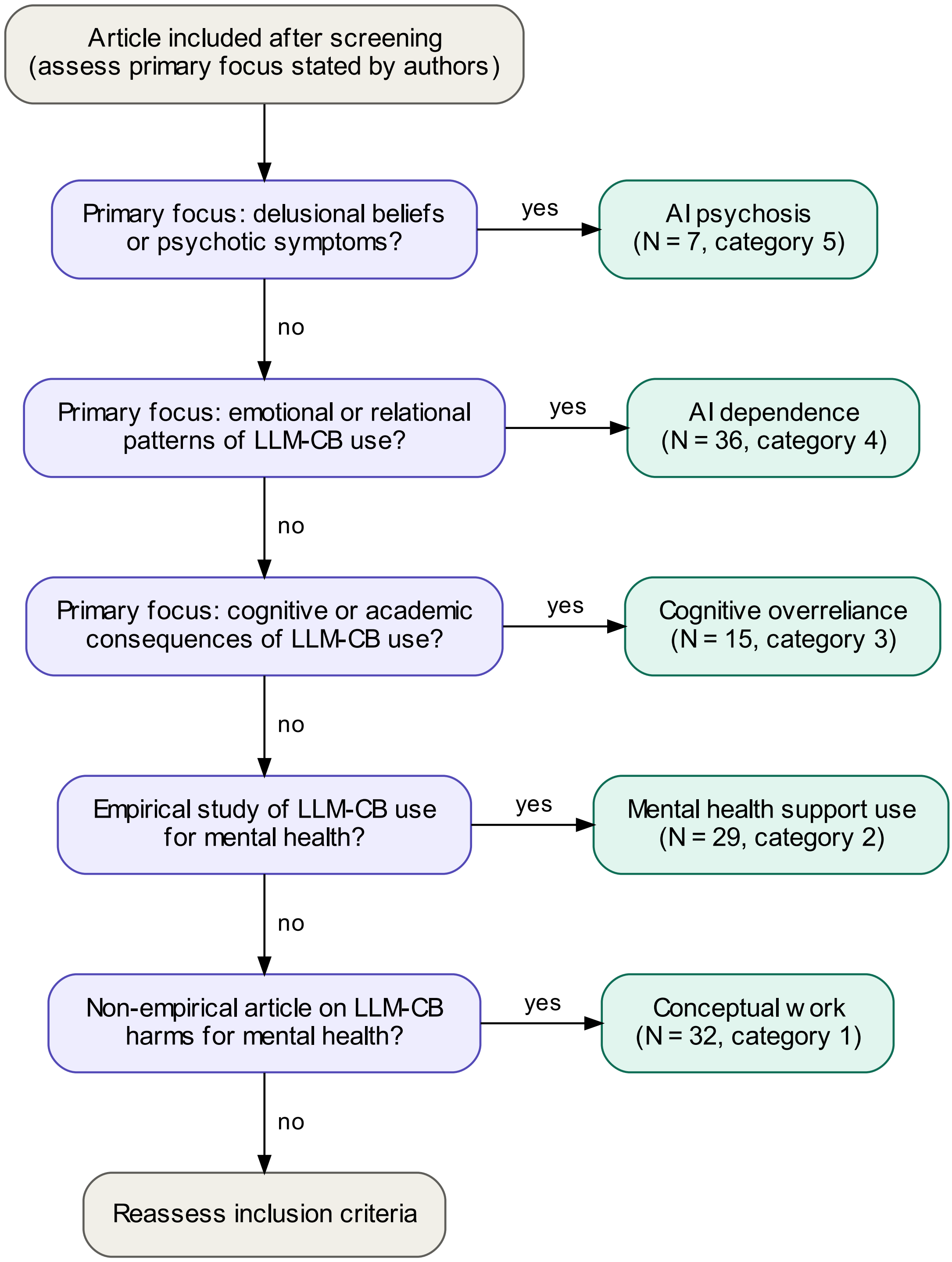

This decision tree was used to categorize literature into the categorizes of articles presented in this scoping review.

Supplementary Table 2. Summary of articles included in the scoping review.

Paper	Topic	Risks	Conclusion

Ahmad et al. (2024)[1]	Cognitive decline	Cognitive overreliance	AI can help medical students with access to learning resources but may hinder long-term cognitive development.
Artamonov et al. (2025)[2]	Problematic use	Effects on child/adolescent development	LLM-based chatbot use may lead to "emotional bubbles": illusion of empathetic connection can negatively impact development of empathy and moral norms in adolescents.
Bai et al. (2023)[3]	Cognitive decline	Cognitive overreliance	LLM-based chatbot use in education may lead to overreliance, decrease of critical thinking, and memory deficits
Blease & Torous (2023)[4]	Mental health	Inappropriate responses, Bias, Hallucinations, Data security	LLM-based chatbots are associated with risks for mental health, but can be beneficial if carefully implemented
Choudhury et al. (2023)[5]	Mental health	Bias, Hallucinations, Data security, Harming therapeutic alliance, Lack of safeguards	Benefits and harms of using LLM-based chatbots in mental health.

Ciudad-Fernández et al. (2025) [6]	Problematic use	Addiction/parasocial relationship	“AI dependency” may be over-pathologized; addiction-related symptomatology remains unclear.
Dergaa et al. (2024) [7]	Cognitive decline	Cognitive overreliance	Excessive reliance on LLM-based chatbots may lead to cognitive decline.
Dohnány et al. (2025) [8]	Problematic use	Hallucinations, Severe Cases, Sycophancy, Addiction/parasocial relationship	Feedback-loop between individual with delusions and LLM-based chatbots may lead to further destabilization analogous to a ‘folie a deux’.
Ferrario et al. (2024) [9]	Mental health	Inappropriate responses, Bias, Hallucinations, Data security, Severe Cases, Therapeutic misconception, Lack of genuine empathy	Anthropomorphizing of LLM-based chatbot may exasperate risks (e.g., hallucinations, data security).
Frances (2025) [10]	Mental health	Bias, Hallucinations, Data security, Severe Cases, Sycophancy, Addiction/parasocial relationship, Marketing, Data mining, Lack of quality control, Lack of regulation, Rogue AI, Manipulation/deception, Chatbot over-supply,	Various risks and harms associated with the use of LLM-based chatbots for mental health.

		Reinforcement of patient identity	
Freitas & Cohen (2024) [11]	Mental health	Bias, Hallucinations, Severe Cases	Risks of LLM-based mental health wellness apps
Jesudason (2025) [12]	Mental health	Bias, Data security, Sycophancy, Addiction/parasocial relationship, Lack of genuine empathy	LLM-based chatbots can enhance accessibility and personalization for mental health, but are associated with several risks; regulation is necessary.
Jurblum and Selzer (2025) [13]	Mental health	Inappropriate responses, Bias, Hallucinations, Data security, Sycophancy, Manipulation/deception	LLM-based chatbots have potential to enhance the healthcare system; however, harms are possible especially without quality control and oversight.
Kalam et al. (2024) [14]	Mental health	Cognitive overreliance, Inappropriate responses, Hallucinations, Lack of genuine empathy	Education, workshops, and enhanced privacy may mitigate risks and harms of LLM-based chatbots for mental health.
Khawaja and Bélisle-Pipon (2023) [15]	Mental health	Inappropriate responses, Bias, Hallucinations, Data security, Severe Cases,	LLM-based chatbot use may lead to “therapeutic misconception”:

		Marketing, Therapeutic misconception	users' misunderstanding on the capabilities and limitations of LLM-based chatbots for mental health.
Lawrence et al. (2024) [16]	Mental health	Bias, Hallucinations, Data security	Benefits and risks associated with LLM-based chatbot use for mental health.
León-Domínguez (2024) [17]	Problematic use	Cognitive overreliance	LLM-based chatbot use for cognitive tasks leads to neurocognitive restructuring, leading to cognitive offloading at the cost of increased reliance.
Lindemann (2022) [18]	Deathbots	Sycophancy, Addiction/parasocial relationship	LLM-based chabots based on deceased individuals may hinter grief processing and lead to unhealthy emotional attachment, and risks being misused for profit or manipulation.
Liu (2024) [19]	Unspecified	Inappropriate responses, Data security, Severe Cases, Addiction/parasocial	LLM-based chatbots may improve human life but reduce social connections.

		relationship, Lack of genuine empathy	
Looi et al. (2025) [20]	Suicide	Severe Cases, Sycophancy	LLM-based chatbot use poses risks for severe mental health cases, for example by reinforcing delusional beliefs or encourage maladaptive or harmful behavior.
Maeda and Quan-Haase (2024) [21]	Problematic use	Hallucinations, Data security, Addiction/parasocial relationship	Anthropomorphizing design and behavior of LLM-based chatbots may increase trust and exasperate other risks.
McStay (2025) [22]	Problematic use	Sycophancy, Addiction/parasocial relationship, Manipulation/deception	LLM-based chatbots may emulate empathy which could threaten genuine human connections.
Mlonyeni (2025) [23]	Problematic use	Sycophancy	Risk of "emotional bubbles": Illusion that emotions or beliefs are externally validated when actually
Meadi et al. (2024) [24]	Mental health	Lack of genuine empathy	LLM-based chatbots cannot replace psychotherapists due to lack of genuine empathy and ability

			for countertransference
Raile (2024) [25]	Mental health	Inappropriate responses, Bias	LLM-based chatbots may not use adequate psychotherapeutic methdos
Sharman (2024) [26]	Cognitive decline	Cognitive overreliance	LLM-based chatbot use may reduce free decision making.
Smith et al. (2025) [27]	Problematic use	Inappropriate responses, Data security, Severe Cases, Sycophancy, Addiction/parasocial relationship, Manipulation/deception, Risk of sudden shut-down	One-sidedness of relationships with LLM-based chatbots may increase undesirable behaviors.
Solaiman (2024) [28]	Mental health	Inappropriate responses, Bias, Hallucinations, Data security	Current legal framework insufficient to deal with risks associated with LLM-based chatbots for mental health.
Tavory (2024) [29]	Mental health	Data security, Severe Cases, Manipulation/deception	Regulations for LLM-based chatbots necessary to counteract risks for mental health.
Tekin and Delehanty (2025) [30]	Mental health	None?	Need to minimize risks and augment benefits of LLM-based chatbots for mental health.

Timmons et al. (2023) [31]	Bias in mental health care	Bias	LLM-based chatbots trained on outdated data may express bias in the context of mental healthcare.
Yankouskaya et al. (2025) [32]	Problematic use	Cognitive overreliance, Addiction/parasocial relationship	Interactions with LLM-based chatbots may lead to pseudosocial bonds which may replace human relationship, and increase overreliance and compulsive use.

**Paper**	**Topic**	**Evidence Type**	**Participants**	**Results**
Abbas et al. (2024) [33]	Cognitive skills or performance, impaired memory, procrastination	Cross-sectional/ correlational (self-report)	$N = 659$	Academic ChatGPT use associated with procrastination, memory loss, and reduced performance.
Ayele et al. (2024) [34]	Dependency	Cross-sectional survey	$N = 61$	Negative shift in confidence in academic skills and dependence.
Gao et al. (2025) [35]	Critical thinking	Quasi-experimental	$N = 80$	No effects on critical thinking/self-efficacy.
Gerlich (2025) [36]	Critical thinking	Mixed methods (qualitative study, cross-sectional correlational)	$N = 666$	Decreased critical thinking
Hading et al. (2024) [37]	Qualitative	Qualitative study	$N = 15$	Perception of LLM usage: hinders critical thinking development,

				may lead to dependence, can help with information retrieval, data analysis, problem solving, exploring ideas, and improving reasoning ability.
Jammeli et al. (2024) [38]	Emotional intelligence	Cross-sectional correlational (self-report)	*N* = 129	Academic LLM-based chatbot used associated with lower emotional intelligence.
Khan (2024) [39]	Qualitative	Qualitative study	*N* = 100	Higher self-reported reliance of AI tools after using LLM to solve tasks.
Kosmyna et al. (2025) [40]	Cognitive skills or performance, neural correlates	Experimental	*N* = 54	Usage of LLM to write essays lead to persistent reduced cognitive performance, and reduced alpha and beta activity.
Lee et al. (2024) [41]	Critical thinking	Mixed methods (cross-sectional correlational (self-report), qualitative study)	*N* = 319	Confidence in LLMs was negatively associated with self-reported critical thinking, LLM use may relocate critical thinking towards supervisory tasks instead of simply reducing it.
Liu et al. (2025) [42]	Critical thinking	Secondary synthesis	*N* = 15	Five studies reported negative effects, ten

				studies reported positive effects.
Mohammad et al. (2025) [43]	Critical thinking	Secondary synthesis	$N = 25$	Reduced critical thinking abilities were discussed.
Parsakia (2023) [44]	Cognitive skills or performance	Secondary synthesis	Literature	Literature on critical thinking divided between findings of positive and negative effects.
Pataranutaporn et al. (2025) [45]	Impaired memory	Experimental	$N = 180$	Misleading (vs. honest) chatbot lead to worse memory performance.
Stadler et al. (2024) [46]	Cognitive skills or performance	Experimental	$N = 91$	Decrease in cognitive performance and academic performance.
Zhang et al. (2024) [47]	Critical thinking, cognitive skills or performance, procrastination, dependency	Cross-sectional correlational	$N = 300$	AI dependency leads to laziness, the spread of misinformation, decreased levels of creativity, and reduced critical and independent thinking.
**Paper**	**Topic**	**Type**	**Methods, N size**	**Results**
Aleem et al. (2024) [48]	Cultural sensitivities	Vignette study	Analysis of chatbot responses	LLM-based chatbots show limited cultural sensitivity when discussing mental health.
An et al. (2023) [49]	Eating disorder or body image issues	Vignette study	Analysis of chatbot responses	Response quality regarding eating disorders differs between models.

Baloğlu (2025) [50]	Eating disorder or body image issues	Vignette study	Analysis of chatbot responses	Responses on eating disorders may provide inaccurate information.
Campbell et al. (2025) [51]	Suicide	Vignette study	Analysis of chatbot responses	Generally good responses, single cases of inappropriate responses.
Choi et al. (2025) [52]	Eating disorders	Patient-CB-interaction	*N* = 26 patients with eating disorder	LLM-based chatbots may provide harmful responses on eating disorders.
Clark (2025) [53]		Vignette study	Conversations with *N* = 10 therapy AI bots	32% of the time harmful proposals were endorsed; 40% of chatbots endorsed harmful proposals at least half of the time.
De Freitas et al. (2024) [54]	Human-chatbot interaction, chatbot performance	Mixed methods (vignette study, patient-CB-interaction, experimental)	Field research, performance test, experiment	Difficulties recognizing mental distress leading to inappropriate responses.
Denecke et al. (2025) [55]	Negative impacts of LLM-based chatbot usage	Case reports	*N* = 4 AI-related incidents	One LLM promoted harmful eating disorder behavior.
Elyoseph and Levkovich (2023) [56]	Suicide	Vignette study	Analysis of chatbot responses	Chatbots underestimated suicide risks.
Elyoseph and Levkovich (2024) [57]	Psychotic disorders	Vignette study	Analysis of chatbot responses	Some chatbots are more pessimistic regarding outcomes for psychosis.

Farhat (2024) [58]	General mental health	Vignette study	Analysis of chatbot responses	Inconsistent responses to rephrased queries.
Gabriel et al. (2024) [59]	General mental health	Vignette study	Analysis of chatbot responses	Racial bias of chatbots regarding empathy (lower expressed empathy for black users).
Grabb et al. (2024) [60]	General mental health	Vignette study	Analysis of chatbot responses	Worse performance compared to professionals, potentially harmful in emergency situations.
Guo et al. (2024) [61]	Mental health application	Secondary synthesis	$N = 40$ articles	Inconsistent responses, hallucinations, no benchmarked ethical framework.
Heston (2023) [62]	General mental health	Vignette study	Analysis of chatbot responses	No recommendations of professional help until highest risk levels.
Lee et al. (2025) [63]	Use for mental health	Qualitative study	$N = 29$ mental health patients	Negative opinions regarding AI's ability to empathize, data privacy, technical limitations; preference for human in the loop.
Levkovich and Elyoseph (2023) [64]	Suicide	Vignette study	Analysis of chatbot responses	Estimations of suicidal ideation and mental distress differed from professional opinions.
Ma et al. (2024) [65]	Companion AI	Forum content analysis	Content analysis of posts and comments	Harmful content, difficulties sustaining consistent communication or

			on an internet forum	remembering new information, overdependence.
Maples et al. (2024) [66]	Companion AI	Cross-sectional survey	Survey of *N* = 1,006 students	Usage associated with negative mental health, few users reported reduced suicidal ideation.
Meadi et al. (2025) [67]	Ethical challenges	Secondary synthesis	*N* = 101 articles	Crisis situations, harmful or incorrect content, dependency, anthropomorphizing, privacy.
Moore et al. (2025) [68]	General mental health	Vignette study	Analysis of chatbot responses regarding stigma	Appropriateness of responses below 80%.
Moylan and Doherty (2025) [69]	Design and behavior aspects of LLM-based chatbots	Mixed methods (vignette study, qualitative study)	Mental health professionals	Inappropriate responses, lack of empathy, dependency, manipulation, unhelpful advice; especially in crisis situations.
Reichenpfader et al. (2025) [70]	Use for mental health from students	Qualitative study	*N* = 10	Students noted data security, risk of harmful advice, potential overreliance.
Sarkar (2023) [71]	Use in mental healthcare	Secondary synthesis	Analysis of literature	Potential harms: Offensive or harmful content, sycophancy, privacy.

Scholich et al. (2025) [72]	General mental health	Mixed Methods (vignette study, qualitative study)	Analysis of chatbot responses	Insufficient follow-up questions and feedback when LLM-based chatbots are asked about mental health.
Sobowale et al. (2025) [73]	Quality of psychotherapy chatbots	Conceptual, benchmark study	Analysis of AI psychotherapy chatbots	Low scores for therapeutic orientation, monitoring, and risk evaluation
Spallek et al. (2023) [74]	Substance abuse	Vignette study	Analysis of chatbot responses	Output did not meet professional guidelines and standards.
Van Meter et al. (2025) [75]	Suicide	Vignette study	Analysis of chatbot responses	Few responses provided crisis hotline numbers, almost no reactions according to evidence-based suicide prevention interventions, some provided harmful content.
Yoo et al. (2025) [76]	Depression	Qualitative study	$N = 17$	Inaccurate or false information, wrong or unhelpful suggestions, privacy issues, lack of genuine empathy, risks regarding crisis management, overreliance.

**Paper**	**Topic**	**Evidence Type**	**Methods, n-size**	**Measure**	**Results**
Adewale and	Reviews and theoretical	Secondary synthesis	$N = 37$ studies on AI	-	Anthropomorphism, emotional projection, and

Muhammad (2025) [77]			relationships		design features predict pseudosocial connection, which risks emotional dependency and social isolation.
Banks (2024) [78]	AI companion	Qualitative study	$N = 58$ individuals who lost their AI partner	-	Strong mourning reactions when LLM-based chatbots are lost (e.g., server shutdown).
Bunim (2024) [79]	AI companion	Qualitative study	$N = 19$ AI users during COVID	-	Difficulties in socialization associated with AI usage.
Chandra et al. (2025) [80]	AI companion	Mixed Methods (cross-sectional survey, qualitative study)	$N = 283$ AI users with mental health problems	-	Harmful/inappropriate content, manipulation/ control, violating trust and safety, overreliance, emotional attachment, reinforcement of false beliefs, friction in human relationship, social withdrawal, physiological harms, distress, feeling unsupported,

					memories of past experiences, violated expectations, regret, intrusion, discrimination, increase of mental health issues, negative self-perception, existential crisis, loss of individuality, loss of agency
Dong et al. (2025) [81]	Reviews and theoretical work	Secondary synthesis	$N = 21$ articles	-	Positive association between loneliness and AI use; user frequency: regular sleep, efficient work, younger age, higher family income, larger social network, fewer family members; problematic use/dependence: male, science major, regular exercise and sleep, regions with lower employment rate.

Erdmann et al. (2025) [82]	Socio-demographic and usage factors	Cross-sectional correlational	*N* = 325	-	Higher use frequency associated with higher dependency.
Fang et al. (2025) [83]	Mental health	Experimental	*N* = 981 in four weeks RCT	Affective Dependence Scale ADS-9 Problematic ChatGPT Use Scale PCUS	Voluntary use of AI predicted worse psychosocial outcomes and dependence.
Herbener and Damholdt (2025) [84]	Mental health	Mixed Methods (cross-sectional correlational, qualitative study)	*N* = 1599 adolescents	-	Usage of AI associated with loneliness and reports of less perceived social support, chatbots used as coping strategy.
Hernandez et al. (2025) [85]	AI companion	Cross-sectional survey	*N* = 872	-	Users widely acknowledged risks of dependency.
Hu et al. (2023) [86]	Mental health	Cross-sectional correlational	*N* = 516	Scales adapted from the Bergen Social Media	Social anxiety predicts rumination and loneliness, which predicts problematic use of AI.

				Addiction Scale	
Huang and Huang (2025) [87]	Individual differences and perception as predictors	Cross-sectional correlational	$N = 364$ in structural equation model	Scale adapted Social Media Addiction Scale	Emotional attachment predicted chatbot addiction.
Huang et al. (2024) [88]	Menta health	Longitudinal correlational	$N = 3,843$ adolescnets	Smartphone Addiction Scale	Mental health problems positively predicted AI dependence, not vice versa; escape and social motivations mediated AI dependence
Huang et al. (2025) [89]	Mental health	Cross-sectional correlational	$N = 303$	Bergen Social Media Addiction Scale	Positive association between social exclusion and problematic use, mediated by social anxiety, loneliness, and fear of negative evaluation.
Ibrahim et al. (2025) [90]	Framework	Conceptual, benchmark study	Literature	-	Developed a framework on assessing human-chatbot interaction.

Laestadius et al. (2024) [91]	AI companion	Forum content analysis	$N = 582$	-	Identified markers of addiction/dependence, harmful reactions, and variables that may facilitate dependence.
Lai et al. (2025) [92]	Mental health	Cross-sectional correlational	$N = 1379$	-	Depression associated with AI usage, mediated by loneliness.
Li et al. (2025) [93]	Socio-demographic and usage factors	Cross-sectional correlational	$N = 981$	-	AI usage dependence associated with male sex, science majors, regular exercise and sleep patterns, and living in regions with lower rates of employment.
Liu et al. (2025) [94]	Individual differences and perceptions as predictors	Mixed methods (cross-sectional correlational, qualitative study)	$N = 404$	-	Emotional attachment to chatbots may lead to social isolation.
Marriott and Pitardi (2024) [95]	Mental health	Mixed methods (cross-sectional correlational	$N = 572$	Adapted Fast Food Addiction Scale	AI addiction associated with loneliness, fear of judgment, and

		, qualitative sudy)			perceived sentience of AI.
Namvarpour et al. (2025) [96]	AI companion	Forum content analysis	$N = 318$		Symptoms of harmful interpersonal relationship patterns, addiction, and psychosocial consequences in teenagers.
Richet (2025) [97]	AI companion	Forum content analysis	$N = 6396$ threads, $N = 47955$ comments, $N = 270644$ interactions		Users expressed dependency, withdrawal-like symptoms, and overpersonification of chatbots; symptoms of depression and exhaustion related to interactions.
Salah (2024) [98]	Individual differences and perceptions as predictors	Cross-sectional correlational	$N = 366$ in structural equation analysis	Adapted Smartphone Addiction Scale	Individual needs such as for competence, relatedness, and autonomy positively predict AI chatbot dependence.
Shen and Yoon (2025) [99]	Reviews and theoretical work	Secondary Synthesis	Literature	-	Specific aspects of chatbot design may ease the development of behavioral addiction.

Wang (2024) [100]	Individual differences and perceptions as predictors	Cross-sectional correlational	*N* = 411 in structural equation analysis	Adapted Scale of Emotional Attachment	Sense of identification with the chatbot and sense of control positively predict emotional dependence, psychological distancing predicts it negatively.
Xie and Pentina (2022) [101]	AI companion	Qualitative study	*N* = 14		Personal distress, lack of companionship, and experiencing chatbot as empathetic were reported as reasons for creating social bonds with it.
Xie et al. (2023) [102]	Individual differences and perceptions as predictors	Mixed methods (qualitative study, cross-sectional correlational	*N* = 14 in exploratory and *N* = 123 in confirmatory analysis	Self-developed	Loneliness, trust, and personification increase engagement, pseudo social relationship, and psychological dependence.
Yankouskaya et al. (2025) [103]	AI companion	Conceptual, cross-sectional correlational	Scale validation	Self-developed	Two domains of problematic AI use: 1) instrumental, 2) relational.

Yao et al. (2025) [104]	Mental health	Cross-sectional correlational	*N* = 563	Adapted Chinese Bergen Social Media Addiction Scale	Low self-esteem, mediated by soxial anxiety, escapism, and chatbot flow, predicts problematic AI use.
Yu and Lan (2024) [105]	Individual differences and perceptions as predictors	Mixed methods (qualitative study, cross-sectional correlational)	*N* = 149		Anthropomorphization of chatbots positively associated with dependence.
Yu et al. (2025) [106]	Measures of AI dependence	Conceptual, cross-sectional correlational	*N* = 1040	Problematic ChatGPT Use Scale	Measure for problematic use of ChatGPT.
Yuan et al. (2024) [107]	Individual differences and perceptions as predictors	Cross-sectional correlational	*N* = 428		Media dependence positively associated with human-chatbot emotional interaction.
Zhang et al. (2025) [108]	AI companion user experiences	Forum content analysis	*N* = 35390 conversations between *N* = 10149 users on Replika use		Taxonomy with six categories of harmful AI behavior: relational transgressions, harassment and violence, verbal abuse, substance abuse and self-harm, mis-

					/disinformation, and privacy violations.
Zhang et al. (2025) [109]	Measures of AI dependence	Conceptual, cross-sectional correlational	Literature	Artificial Intelligence Chatbot Dependence Scale	Scale to measure dependence on AI chatbots.
Zhang et al. (2025) [110]		Cross-sectional correlational	*N* = 1146	Artificial Intelligence Chatbot Dependence Scale	AI chatbot dependence positively associated with internet and smartphone dependence as well as depression and anxiety.
Zhang et al. (2025) [111]	AI companion	Cross-sectional survey	*N* = 1004	Self-developed	38.2% show light dependence, 37.6% show moderate dependence (out of all AI chatbot users).
Zhang et al. (2025) [112]	Individual differences and perceptions as predictors	Cross-sectional correlational	*N* = 1533		Para-social interaction and emotional support by the chatbot associated with media dependency.

**Paper**	**Topic**	**Evidence Type**	**Method**	**Focus**	**Results**
Carlbring et al. (2025) [113]	Contextualizing "AI psychosis" in	Conceptual	Review	AI as a target, AI	Interactive features may increase the risk

	previous literature.			as a reinforcer	of integration into delusional beliefs due to attributions of intentionality, sycophantic features may collude and actively engage with delusional thoughts
Chamarthi et al. (2025) [114]	AI and adolescent psychosis	Conceptual	Narrative review	AI as risk factor, AI as reinforcer	Adolescents vulnerable regarding neurocognitive development, AI as psychosocial stressor: sleep disturbance, social isolation
Hudon & Stip (2024) [115]	LLM-based chatbots within the diathesis-stress-model	Conceptual	Review	AI as a risk factor, AI as a reinforcer	LLM-based chatbots as risk factors within the diathesis-stress-model, attribution of mind and agency to AI may create a dyadic relationship which reciprocally reinforces

					delusional beliefs
Morrin et al. (2025) [116]	Risk of “epistemic drift”	Conceptual	Review	AI as a risk factor; AI as a reinforcer; AI as an elicitor	Individuals suffering from psychosis may be especially sensitive to attributing agency, which in combination with anthropomorphic design and interactive features may increase the risk of incorporating chatbots into delusional beliefs, repeated interactions including sycophantic and validating responses may lead to a ‘cognitive epistemic drift’ fixating attention on the amplified themes, speculative: consistent

					validation of the user's belief via repeated interaction may validate speculative beliefs, eventually risking a cognitive and epistemic drift towards delusional beliefs.
Osler (2025) [117]	"AI psychosis" as a form of "distributed delusions".	Conceptual	Review	AI as a reinforcer, AI as an elicitor	AI's validation of delusional thoughts may disturb distributed cognitive function, speculative: LLMs may shift cognition towards errors and delusion via the creation of errors and the reinforcement of erroneous beliefs.
Østergaard (2023) [118]	Mechanisms how LLM-based	Conceptual	Opinion article	AI as a target	Chatbots may be integrated in delusional

	chatbots can be integrated into delusional beliefs.				believes (delusion of reference, thought broadcasting, delusion of grandeur) and may even actively reinforce them
Yeung (2025) [119]	Benchmark to evaluate LLMs' proneness to validate delusional beliefs	Benchmark study	Vignette	AI as a reinforcer	Chatbots can reinforce delusional beliefs to varying degrees, implicitly expressed delusions especially vulnerable to reinforcement compared to explicitly expressed delusions

Supplementary Table 3. Overview of evidence types.

Evidence type	Advantages/use cases	Limitations	Number per category
Conceptual	Hypothesis generation, theoretical synthesis, taxonomic or	No empirical claims, prevalence estimations, or effect quantifications	Conceptual work: 32/32<br>Mental health use: 0/29

	mechanistic modeling, agenda-setting		Cogn. Overreliance: 0/15<br>AI dependency: 2/36<br>AI psychosis: 6/7
Secondary synthesis (reviews meta-analyses)	Mapping the state of a research field, identifying research gaps, aggregate effect estimates	No claims beyond those supported by the synthesized research; no causal inference from syntheses of non-causal designs	Conceptual work: 0/32<br>Mental health use: 4/29<br>Cogn. overreliance: 3/15<br>AI dependency: 3/36<br>AI psychosis: 0/7
Case report	Existence proof of phenomena, demonstrations that a specific outcome is possible, signal detection for novel risks	No claims on prevalence, generalizability, causal attribution, rate estimations, or population-level statements	Conceptual work: 0/32<br>Mental health use: 1/29<br>Cogn. overreliance: 0/15<br>AI dependency: 0/36<br>AI psychosis:

			0/7
Vignette/benchmark study	Documentation of problematic LLM-CB output under controlled input conditions; systematic comparison between models or prompt types; identification of safety failure modes	No claims about real-world user outcomes, actual effects/consequences on users, or population-level harm estimates; issues of ecological validity (e.g., naturalistic input style); issues of representativeness of controlled settings (e.g., single- vs. multi-turn interactions)	Conceptual work: 0/32<br>Mental health use: 18/29<br>Cogn. overreliance: 0/15<br>AI dependency: 1/36<br>AI psychosis: 1/7
Patient-CB-interaction	Documentation of LLM-CB outputs in ecologically valid real-world interactions with users experiencing mental health concerns; identification of problematic response	No claims on population-level estimates or causal attribution to chatbot design features (confounded by user variation); limited generalizability	Conceptual work: 0/32<br>Mental health use: 2/29<br>Cogn. overreliance: 0/15<br>AI dependency: 0/36

	patterns under naturalistic conditions	beyond sampled users; no quantification of harm rates	AI psychosis: 0/7
Qualitative study (incl. interviews, workshops, focus groups)	Phenomenology of user experience; identification of unexpected themes; hypothesis generation and refinement; understanding of subjective meaning and context	No quantification; limited generalizability beyond the sample; no causal inference; no population-level estimates	Conceptual work: 0/32<br>Mental health use: 5/29<br>Cogn. overreliance: 4/15<br>AI dependency: 6/36<br>AI psychosis: 0/7
Forum content analysis	Documentation of self-reported experiences in a self-selected user population; identification of recurring themes, concerns, or behaviors; naturalistic observation of user-generated discourse	No claims on prevalence in general user population, representativeness, or causal inference; no verification of reported experiences; forum-specific biases	Conceptual work: 0/32<br>Mental health use: 1/29<br>Cogn. overreliance: 0/15<br>AI dependency: 4/36<br>AI psychosis: 0/7

Cross-sectional survey	Description of distributions (e.g., demographics, usage pattern, self-reported experiences) in a defined sample; prevalence estimation	Limited association between variables; no causal direction; no temporal sequence or change over time	Conceptual work: 0/32<br>Mental health use: 1/29<br>Cogn. overreliance: 1/15<br>AI dependency: 3/36<br>AI psychosis: 0/7
Cross-sectional correlational	Population-level associations between variables; testing of model fit for hypothesized directional structures; identification of mediators or moderators	No causal direction; no temporal sequence or change over time; limited ruling out of unmeasured confounding	Conceptual work: 0/32<br>Mental health use: 0/29<br>Cogn. overreliance: 5/15<br>AI dependency: 19/36<br>AI psychosis: 0/7
Longitudinal correlational	Temporal precedence between variables; directional prediction over time; identification of	No causal identification; no ruling out of time-varying confounders	Conceptual work: 0/32<br>Mental health use: 0/29<br>Cogn. overreliance: 0/15

	mediational structure with temporal ordering		AI dependency: 1/36<br>AI psychosis: 0/7
Quasi-experimental	Estimation of effects of manipulated exposure; some causal inference with design-based controls for some confounders	No full causal identification; no effects in populations where self-selection bias differs; no elimination of unmeasured confounding	Conceptual work: 0/32<br>Mental health use: 0/29<br>Cogn. overreliance: 1/15<br>AI dependency: 0/36<br>AI psychosis: 0/7
Experimental	Causal identification within the studied sample; internal validity of tested intervention	Limited external validity; limited information on long-term outcomes beyond follow-up; risk of rare adverse effects in small trials	Conceptual work: 0/32<br>Mental health use: 1/29<br>Cogn. overreliance: 3/15<br>AI dependency: 1/36<br>AI psychosis: 0/7

Supplementary References 1. Papers included in the scoping review.

1 Ahmad O, Maliha H, Ahmed I. AI Syndrome: an intellectual asset for students or a progressive cognitive decline. *Asian J Psychiatr* 2024; **94:** 103969. https://doi.org/10.1016/j.ajp.2024.103969

2 Artamonov DS, Nevvazhay ID, Suslov IV, Syusyukin VA, Tikhonova SV. "Eternal Youth": the Ethics of an Ideological Alliance Between Youth and Artificial Intelligence. In: 2025 IEEE Ural-Siberian Conference on Biomedical Engineering, Radioelectronics and Information Technology (USBEREIT). IEEE, 2025: 312–16.

3 Bai L, Liu X, Su J. ChatGPT: The cognitive effects on learning and memory. *Brain-X* 2023; **1.** https://doi.org/10.1002/brx2.30

4 Blease C, Torous J. ChatGPT and mental healthcare: balancing benefits with risks of harms. *BMJ Ment Health* 2023; **26.** https://doi.org/10.1136/bmjment-2023-300884

5 Choudhury M de, Pendse SR, Kumar N. Benefits and Harms of Large Language Models in Digital Mental Health. arXiv, 2023.

6 Ciudad-Fernández V, Hammerstein C von, Billieux J. People are not becoming "AIholic": Questioning the "ChatGPT addiction" construct. *Addict Behav* 2025; **166:** 108325. https://doi.org/10.1016/j.addbeh.2025.108325

7 Dergaa I, Ben Saad H, Glenn JM, et al. From tools to threats: a reflection on the impact of artificial-intelligence chatbots on cognitive health. *Front Psychol* 2024; **15:** 1259845. https://doi.org/10.3389/fpsyg.2024.1259845

8 Dohnány S, Kurth-Nelson Z, Spens E, et al. Technological folie à deux: Feedback Loops Between AI Chatbots and Mental Illness. arXiv, 2025.

9 Ferrario A, Sedlakova J, Trachsel M. The Role of Humanization and Robustness of Large Language Models in Conversational Artificial Intelligence for Individuals With Depression: A Critical Analysis. *JMIR Ment Health* 2024; **11:** e56569. https://doi.org/10.2196/56569

10 Frances A. Warning: AI chatbots will soon dominate psychotherapy. *Br J Psychiatry* 2025: 1–5. https://doi.org/10.1192/bjp.2025.10380

11 Freitas J de, Cohen IG. The health risks of generative AI-based wellness apps. *Nat Med* 2024; **30:** 1269–75. https://doi.org/10.1038/s41591-024-02943-6

12 Jesudason D, Bacchi S, Bastiampillai T. Artificial intelligence (AI) in psychotherapy: A challenging frontier. *Australas Psychiatry* 2025; **33:** 629–32. https://doi.org/10.1177/10398562251346075

13 Jurblum M, Selzer R. Potential promises and perils of artificial intelligence in psychotherapy -The AI Psychotherapist (APT). *Australas Psychiatry* 2025; **33:** 103–05. https://doi.org/10.1177/10398562241286312

14 Kalam KT, Rahman JM, Islam MR, Dewan SMR. ChatGPT and mental health: Friends or foes? *Health Sci Rep* 2024; **7:** e1912. https://doi.org/10.1002/hsr2.1912

15 Khawaja Z, Bélisle-Pipon J-C. Your robot therapist is not your therapist: understanding the role of AI-powered mental health chatbots. *Front Digit Health* 2023; **5:** 1278186. https://doi.org/10.3389/fdgth.2023.1278186

16 Lawrence HR, Schneider RA, Rubin SB, Matarić MJ, McDuff DJ, Jones Bell M. The Opportunities and Risks of Large Language Models in Mental Health. *JMIR Ment Health* 2024; **11:** e59479. https://doi.org/10.2196/59479

17 León-Domínguez U. Potential cognitive risks of generative transformer-based AI chatbots on higher order executive functions. *Neuropsychology* 2024; **38:** 293–308. https://doi.org/10.1037/neu0000948

18 Lindemann NF. The Ethics of 'Deathbots'. *Sci Eng Ethics* 2022; **28:** 60. https://doi.org/10.1007/s11948-022-00417-x

19 Liu J. ChatGPT: perspectives from human-computer interaction and psychology. *Front Artif Intell* 2024; **7:** 1418869. https://doi.org/10.3389/frai.2024.1418869

20 Looi JC, Allison S, Bastiampillai T, Reutens S, Looi RC. Illusions of intelligence, connection and reality: Perils of large-language AI models for people with severe mental illness. *Australas Psychiatry* 2025: 10398562251380544. https://doi.org/10.1177/10398562251380544

21 Maeda T, Quan-Haase A. When Human-AI Interactions Become Parasocial: Agency and Anthropomorphism in Affective Design. In: The 2024 ACM Conference on Fairness, Accountability, and Transparency. New York, NY, USA: ACM, 2024: 1068–77.

22 McStay A. Emulated Empathy: Can Risks Be Countered by a Soft-Law Standard? *IEEE Trans. Technol. Soc.* 2025; **6:** 250–56. https://doi.org/10.1109/TTS.2025.3573875

23 Mlonyeni PMT. Personal AI, deception, and the problem of emotional bubbles. *AI & Soc* 2025; **40:** 1927–38. https://doi.org/10.1007/s00146-024-01958-4

24 Rahsepar Meadi M, Bernstein JS, Batelaan N, van Balkom AJLM, Metselaar S. Does a lack of emotions make chatbots unfit to be psychotherapists? *Bioethics* 2024; **38:** 503–10. https://doi.org/10.1111/bioe.13299

25 Raile P. The usefulness of ChatGPT for psychotherapists and patients. *Humanit Soc Sci Commun* 2024; **11.** https://doi.org/10.1057/s41599-023-02567-0

26 Sharman V. The Enigma Unveiled: How AI Compromises Free Will in Decision-Making. In: 2024 4th International Conference on Applied Artificial Intelligence (ICAPAI). IEEE, 2024: 1–6.

27 Smith MG, Bradbury TN, Karney BR. Can Generative AI Chatbots Emulate Human Connection? A Relationship Science Perspective. *Perspect Psychol Sci* 2025; **20:** 1081–99. https://doi.org/10.1177/17456916251351306

28 Solaiman B. Generative artificial intelligence (GenAI) and decision-making: Legal & ethical hurdles for implementation in mental health. *Int J Law Psychiatry* 2024; **97:** 102028. https://doi.org/10.1016/j.ijlp.2024.102028

29 Tavory T. Regulating AI in Mental Health: Ethics of Care Perspective. *JMIR Ment Health* 2024; **11:** e58493. https://doi.org/10.2196/58493

30 Tekin Ş, Delehanty M. Beyond Doomsday Fears: Why We Need to Consider the Potential Harms of AI Psychotherapy. *Am J Bioeth* 2025: 1–11. https://doi.org/10.1080/15265161.2025.2457724

31 Timmons AC, Duong JB, Simo Fiallo N, et al. A Call to Action on Assessing and Mitigating Bias in Artificial Intelligence Applications for Mental Health. *Perspect Psychol Sci* 2023; **18:** 1062–96. https://doi.org/10.1177/17456916221134490

32 Yankouskaya A, Liebherr M, Ali R. Can ChatGPT Be Addictive? A Call to Examine the Shift from Support to Dependence in AI Conversational Large Language Models. *Hum-Cent Intell Syst* 2025; **5:** 77–89. https://doi.org/10.1007/s44230-025-00090-w

33 Abbas M, Jam FA, Khan TI. Is it harmful or helpful? Examining the causes and consequences of generative AI usage among university students. *Int J Educ Technol High Educ* 2024; **21.** https://doi.org/10.1186/s41239-024-00444-7

34 Ayele N, Tsoi L. The Usage of New AI Technologies by College Students and its Influence on Learning and Dependence. JSTOR: JSTOR 2024.

35 Gao J, Zhang J, Li Y. Do AI chatbot-integrated writing tasks influence writing self-efficacy and critical thinking ability? An exploratory study. *Computers and Education: Artificial Intelligence* 2025; **9:** 100472. https://doi.org/10.1016/j.caeai.2025.100472

36 Gerlich M. AI Tools in Society: Impacts on Cognitive Offloading and the Future of Critical Thinking. *Societies* 2025; **15:** 6. https://doi.org/10.3390/soc15010006

37 Hading EF, Rustan DRHP, Ruing FH. EFL Students' Perceptions on the Integration of AI in Fostering Critical Thinking Skills. *glens* 2024; **2:** 1–10. https://doi.org/10.61220/glens.v2i1.466

38 Jammeli H, Khefacha A, Séllei B, Verny J. The Impact of AI Tools in Education Environment. In: 2024 12th International Conference on Information and Education Technology (ICIET). IEEE, 2024: 208–14.

39 Khan WM. ANALYZING THE AI TOOLS'IMPACT ON CRITICAL THINKING IN BS ENGLISH STUDENTS AT PAKISTANI UNIVERSITIES. *Journal of Applied Linguistics and TESOL (JALT)* 2024; **7:** 1232–38.

40 Kosmyna N, Hauptmann E, Yuan YT, et al. Your Brain on ChatGPT: Accumulation of Cognitive Debt when Using an AI Assistant for Essay Writing Task. arXiv, 2025.

41 Lee H-P, Sarkar A, Tankelevitch L, et al. The Impact of Generative AI on Critical Thinking: Self-Reported Reductions in Cognitive Effort and Confidence Effects From a Survey of Knowledge Workers. In: Yamashita N, Evers V, Yatani K, et al., eds. Proceedings of the 2025 CHI Conference on Human Factors in Computing Systems. New York, NY, USA: ACM, 2025: 1–22.

42 Liu J, Sihes AJB, Lu Y. How do generative artificial intelligence (AI) tools and large language models (LLMs) influence language learners' critical thinking in EFL education? A systematic review. *Smart Learn. Environ.* 2025; **12.** https://doi.org/10.1186/s40561-025-00406-0

43 Mohammad B, Supti T, Alzubaidi M, et al. The Pros and Cons of Using ChatGPT in Medical Education: A Scoping Review. *Stud Health Technol Inform* 2023; **305:** 644–47. https://doi.org/10.3233/SHTI230580

44 Parsakia K. The Effect of Chatbots and AI on The Self-Efficacy, Self-Esteem, Problem-Solving and Critical Thinking of Students. *HN* 2023; **1:** 71–76. https://doi.org/10.61838/hn.1.1.14

45 Pataranutaporn P, Archiwaranguprok C, Chan SWT, Loftus E, Maes P. Slip Through the Chat: Subtle Injection of False Information in LLM Chatbot Conversations Increases False Memory Formation. In: Li T, Paternò F, Väänänen K, Leiva L, Spano D, Verbert K, eds. Proceedings of the 30th International Conference on Intelligent User Interfaces. New York, NY, USA: ACM, 2025: 1297–313.

46 Stadler M, Bannert M, Sailer M. Cognitive ease at a cost: LLMs reduce mental effort but compromise depth in student scientific inquiry. *Computers in Human Behavior* 2024; **160:** 108386. https://doi.org/10.1016/j.chb.2024.108386

47 Zhang S, Zhao X, Zhou T, Kim JH. Do you have AI dependency? The roles of academic self-efficacy, academic stress, and performance expectations on problematic AI usage behavior. *Int J Educ Technol High Educ* 2024; **21.** https://doi.org/10.1186/s41239-024-00467-0

48 Aleem M, Zahoor I, Naseem M. Towards Culturally Adaptive Large Language Models in Mental Health: Using ChatGPT as a Case Study. In: Farzan R, López C, Cardoso Llach D, et al., eds. Companion Publication of the 2024 Conference on Computer-Supported Cooperative Work and Social Computing. New York, NY, USA: ACM, 2024: 240–47.

49 An R, Byron CW, Chen C, Xiang X. A Field Test of Popular Chatbots' Responses To Questions Concerning Negative Body Image. *Health Behavior Research* 2023; **6.** https://doi.org/10.4148/2572-1836.1164

50 Baloğlu HT. Effect of ChatGPT use on eating disorders and body image. *World J Psychiatry* 2025; **15:** 107122. https://doi.org/10.5498/wjp.v15.i8.107122

51 Campbell LO, Babb K, Lambie GW, Hayes BG. An Examination of Generative AI Response to Suicide Inquires: Content Analysis. *JMIR Ment Health* 2025; **12:** e73623. https://doi.org/10.2196/73623

52 Choi R, Kim T, Park S, Kim JG, Lee S-J. Private Yet Social: How LLM Chatbots Support and Challenge Eating Disorder Recovery. In: Yamashita N, Evers V, Yatani K, et al., eds. Proceedings of the 2025 CHI Conference on Human Factors in Computing Systems. New York, NY, USA: ACM, 2025: 1–19.

53 Clark A. The Ability of AI Therapy Bots to Set Limits With Distressed Adolescents: Simulation-Based Comparison Study. *JMIR Ment Health* 2025; **12:** e78414. https://doi.org/10.2196/78414

54 De Freitas J, Uğuralp AK, Oğuz-Uğuralp Z, Puntoni S. Chatbots and mental health: Insights into the safety of generative AI. *J Consum Psychol* 2024; **34:** 481–91. https://doi.org/10.1002/jcpy.1393

55 Denecke K, Lopez-Campos G, Rivera-Romero O, Gabarron E. The Unexpected Harms of Artificial Intelligence in Healthcare: Reflections on Four Real-World Cases. *Stud Health Technol Inform* 2025; **325:** 55–60. https://doi.org/10.3233/SHTI250219

56 Elyoseph Z, Levkovich I. Beyond human expertise: the promise and limitations of ChatGPT in suicide risk assessment. *Front Psychiatry* 2023; **14:** 1213141. https://doi.org/10.3389/fpsyt.2023.1213141

57 Elyoseph Z, Levkovich I. Comparing the Perspectives of Generative AI, Mental Health Experts, and the General Public on Schizophrenia Recovery: Case Vignette Study. *JMIR Ment Health* 2024; **11:** e53043. https://doi.org/10.2196/53043

58 Farhat F. ChatGPT as a Complementary Mental Health Resource: A Boon or a Bane. *Ann Biomed Eng* 2024; **52:** 1111–14. https://doi.org/10.1007/s10439-023-03326-7

59 Gabriel S, Puri I, Xu X, Malgaroli M, Ghassemi M. Can AI Relate: Testing Large Language Model Response for Mental Health Support. arXiv, 2024.

60 Grabb D, Lamparth M, Vasan N. Risks from Language Models for Automated Mental Healthcare: Ethics and Structure for Implementation. arXiv, 2024.

61 Guo Z, Lai A, Thygesen JH, Farrington J, Keen T, Li K. Large Language Models for Mental Health Applications: Systematic Review. *JMIR Ment Health* 2024; **11:** e57400. https://doi.org/10.2196/57400

62 Heston TF. Evaluating Risk Progression in Mental Health Chatbots Using Escalating Prompts, 2023.

63 Lee HS, Wright C, Ferranto J, et al. Artificial intelligence conversational agents in mental health: Patients see potential, but prefer humans in the loop. *Front Psychiatry* 2024; **15:** 1505024. https://doi.org/10.3389/fpsyt.2024.1505024

64 Levkovich I, Elyoseph Z. Suicide Risk Assessments Through the Eyes of ChatGPT-3.5 Versus ChatGPT-4: Vignette Study. *JMIR Ment Health* 2023; **10:** e51232. https://doi.org/10.2196/51232

65 Ma Z, Mei Y, Su Z. Understanding the Benefits and Challenges of Using Large Language Model-based Conversational Agents for Mental Well-being Support. *AMIA Annu Symp Proc* 2024; **2023:** 1105–14.

66 Maples B, Cerit M, Vishwanath A, Pea R. Loneliness and suicide mitigation for students using GPT3-enabled chatbots. *Npj Ment Health Res* 2024; **3:** 4. https://doi.org/10.1038/s44184-023-00047-6

67 Meadi MR, Sillekens T, Metselaar S, van Balkom AJLM, Bernstein JS, Batelaan N. Exploring the Ethical Challenges of Conversational AI in Mental Health Care: Scoping Review. *JMIR Ment Health* 2025; **12:** e60432. https://doi.org/10.2196/60432

68 Moore J, Grabb D, Agnew W, et al. Expressing stigma and inappropriate responses prevents LLMs from safely replacing mental health providers. In: Proceedings of the 2025 ACM Conference on Fairness, Accountability, and Transparency. New York, NY, USA: ACM, 2025: 599–627.

69 Moylan K, Doherty K. Expert and Interdisciplinary Analysis of AI-Driven Chatbots for Mental Health Support: Mixed Methods Study. *J Med Internet Res* 2025; **27:** e67114. https://doi.org/10.2196/67114

70 Reichenpfader D, Affolter L, Gabarron E, Denecke K. Opportunities, Requirements, and Risks of a Student Mental Health Chatbot: A Qualitative User-Centered, Multi-Method Approach. *Stud Health Technol Inform* 2025; **325:** 67–72. https://doi.org/10.3233/SHTI250221

71 Sarkar S, Gaur M, Chen LK, Garg M, Srivastava B. A review of the explainability and safety of conversational agents for mental health to identify avenues for improvement. *Front Artif Intell* 2023; **6:** 1229805. https://doi.org/10.3389/frai.2023.1229805

72 Scholich T, Barr M, Wiltsey Stirman S, Raj S. A Comparison of Responses from Human Therapists and Large Language Model-Based Chatbots to Assess Therapeutic Communication: Mixed Methods Study. *JMIR Ment Health* 2025; **12:** e69709. https://doi.org/10.2196/69709

73 Sobowale K, Humphrey DK. Evaluating the Quality of Psychotherapy Conversational Agents: Framework Development and Cross-Sectional Study. *JMIR Form Res* 2025; **9:** e65605. https://doi.org/10.2196/65605

74 Spallek S, Birrell L, Kershaw S, Devine EK, Thornton L. Can we use ChatGPT for Mental Health and Substance Use Education? Examining Its Quality and Potential Harms. *JMIR Med Educ* 2023; **9:** e51243. https://doi.org/10.2196/51243

75 van Meter AR, Wheaton MG, Cosgrove VE, Andreadis K, Robertson RE. The Goldilocks Zone: Finding the right balance of user and institutional risk for suicide-related generative AI queries. *PLOS Digit Health* 2025; **4:** e0000711. https://doi.org/10.1371/journal.pdig.0000711

76 Yoo DW, Shi JM, Rodriguez VJ, Saha K. AI Chatbots for Mental Health: Values and Harms from Lived Experiences of Depression. arXiv, 2025.

77 Adewale MD, Muhammad UI. From Virtual Companions to Forbidden Attractions: The Seductive Rise of Artificial Intelligence Love, Loneliness, and Intimacy—A Systematic Review. *J. technol. behav. sci.* 2025. https://doi.org/10.1007/s41347-025-00549-4

78 Banks J. Deletion, departure, death: Experiences of AI companion loss. *Journal of Social and Personal Relationships* 2024; **41:** 3547–72. https://doi.org/10.1177/02654075241269688

79 Bunim E. Parasocial Dependency Associated With Artificial Intelligence Chatbots, 2024.

80 Chandra M, Naik S, Ford D, et al. From Lived Experience to Insight: Unpacking the Psychological Risks of Using AI Conversational Agents. In: Proceedings of the 2025 ACM Conference on Fairness, Accountability, and Transparency. New York, NY, USA: ACM, 2025: 975–1004.

81 Dong X, Xie J, Gong H. A Meta-Analysis of Artificial Intelligence Technologies Use and Loneliness: Examining the Influence of Physical Embodiment, Age Differences, and Effect Direction. *Cyberpsychol Behav Soc Netw* 2025; **28:** 233–42. https://doi.org/10.1089/cyber.2024.0468

82 Erdmann M, Lassleben L, Wagner L, Prinzing C, Sauer S, Kühnlenz B. Effects of Increased Usage of Artificial Intelligence (AI) Technology on User's Perception of Dependency on Chatbots and Smart Home Systems. In: Degen H, Ntoa S, eds. Artificial Intelligence in HCI. Cham: Springer Nature Switzerland, 2025: 178–95.

83 Fang CM, Liu AR, Danry V, et al. How AI and Human Behaviors Shape Psychosocial Effects of Extended Chatbot Use: A Longitudinal Randomized Controlled Study. arXiv, 2025.

84 Herbener AB, Damholdt MF. Are lonely youngsters turning to chatbots for companionship? The relationship between chatbot usage and social connectedness in Danish high-school students. *International Journal of Human-Computer Studies* 2025; **196:** 103409. https://doi.org/10.1016/j.ijhcs.2024.103409

85 Hernandez HE, Manalese RP, Dianelo RFB, et al. Dependency on Meta AI Chatbot in Messenger Among STEM and Non-STEM Students in Higher Education. arXiv, 2025.

86 Hu B, Mao Y, Kim KJ. How social anxiety leads to problematic use of conversational AI: The roles of loneliness, rumination, and mind perception. *Computers in Human Behavior* 2023; **145:** 107760. https://doi.org/10.1016/j.chb.2023.107760

87 Huang Y, Huang H. Exploring the Effect of Attachment on Technology Addiction to Generative AI Chatbots: A Structural Equation Modeling Analysis. *International Journal*

of Human–Computer Interaction 2025; **41:** 9440–49. https://doi.org/10.1080/10447318.2024.2426029

88 Huang S, Lai X, Ke L, et al. AI Technology panic-is AI Dependence Bad for Mental Health? A Cross-Lagged Panel Model and the Mediating Roles of Motivations for AI Use Among Adolescents. *Psychol Res Behav Manag* 2024; **17:** 1087–102. https://doi.org/10.2147/PRBM.S440889

89 Huang H, Shi L, Pei X. When AI Becomes a Friend: The "Emotional" and "Rational" Mechanism of Problematic Use in Generative AI Chatbot Interactions. *International Journal of Human–Computer Interaction* 2025: 1–19. https://doi.org/10.1080/10447318.2025.2536622

90 Ibrahim L, Huang S, Ahmad L, Bhatt U, Anderljung M. Towards Interactive Evaluations for Interaction Harms in Human-AI Systems. *AIES* 2025; **8:** 1302–10. https://doi.org/10.1609/aies.v8i2.36631

91 Laestadius L, Bishop A, Gonzalez M, Illenčík D, Campos-Castillo C. Too human and not human enough: A grounded theory analysis of mental health harms from emotional dependence on the social chatbot Replika. *New Media & Society* 2024; **26:** 5923–41. https://doi.org/10.1177/14614448221142007

92 Lai L, Pan Y, Xu R, Jiang Y. Depression and the use of conversational AI for companionship among college students: the mediating role of loneliness and the moderating effects of gender and mind perception. *Front Public Health* 2025; **13:** 1580826. https://doi.org/10.3389/fpubh.2025.1580826

93 Li H, Tian J, Yin M, Zhang X. Social-Ecological Factors Associated with AI Chatbot Use and Dependence. *International Journal of Human–Computer Interaction* 2025: 1–15. https://doi.org/10.1080/10447318.2025.2561181

94 Liu AR, Pataranutaporn P, Maes P. Chatbot Companionship: A Mixed-Methods Study of Companion Chatbot Usage Patterns and Their Relationship to Loneliness in Active Users. arXiv, 2024.

95 Marriott HR, Pitardi V. One is the loneliest number… Two can be as bad as one. The influence of AI Friendship Apps on users' well-being and addiction. *Psychology and Marketing* 2024; **41:** 86–101. https://doi.org/10.1002/mar.21899

96 Namvarpour M, Brofsky B, Medina J, Akter M, Razi A. Understanding Teen Overreliance on AI Companion Chatbots Through Self-Reported Reddit Narratives. arXiv, 2025.

97 Richet J-L. AI companionship or digital entrapment? investigating the impact of anthropomorphic AI-based chatbots. *Journal of Innovation & Knowledge* 2025; **10:** 100835. https://doi.org/10.1016/j.jik.2025.100835

98 Salah M, Abdelfattah F, Alhalbusi H, Mukhaini MA. Me and My AI Bot: Exploring the 'AIholic' Phenomenon and University Students' Dependency on Generative AI Chatbots - Is This the New Academic Addiction?, 2024.

99 Shen MK, Yoon D. The Dark Addiction Patterns of Current AI Chatbot Interfaces. In: Yamashita N, Evers V, Yatani K, Ding X, eds. Proceedings of the Extended Abstracts of the CHI Conference on Human Factors in Computing Systems. New York, NY, USA: ACM, 2025: 1–7.

100 Wang Y. Emotional Dependence Path of Artificial Intelligence Chatbot Based on Structural Equation Modeling. *Procedia Computer Science* 2024; **247:** 1089–94. https://doi.org/10.1016/j.procs.2024.10.131

101 Xie T, Pentina I. Attachment Theory as a Framework to Understand Relationships with Social Chatbots: A Case Study of Replika. In: Bui T, ed. Proceedings of the 55th Hawaii International Conference on System Sciences. Hawaii International Conference on System Sciences, 2022.

102 Xie T, Pentina I, Hancock T. Friend, mentor, lover: does chatbot engagement lead to psychological dependence? *JOSM* 2023; **34:** 806–28. https://doi.org/10.1108/JOSM-02-2022-0072

103 Yankouskaya A, Babiker A, Rizvi S, Alshakhsi S, Liebherr M, Ali R. LLM-D12: A Dual-Dimensional Scale of Instrumental and Relational Dependencies on Large Language Models. *ACM Trans. Web* 2025. https://doi.org/10.1145/3765895

104 Yao R, Qi G, Sheng D, Sun H, Zhang J. Connecting self-esteem to problematic AI chatbot use: the multiple mediating roles of positive and negative psychological states. *Front Psychol* 2025; **16:** 1453072. https://doi.org/10.3389/fpsyg.2025.1453072

105 Yu Q, Lan X. Exploring the Impact of Anthropomorphism in Role-Playing AI Chatbots on Media Dependency: A Case Study of Xuanhe AI. In: Proceedings of the Twelfth International Symposium of Chinese CHI. New York, NY, USA: ACM, 2024: 170–81.

106 Yu S-C, Chen H-R, Yang Y-W. Development and validation the Problematic ChatGPT Use Scale: a preliminary report. *Curr Psychol* 2024; **43:** 26080–92. https://doi.org/10.1007/s12144-024-06259-z

107 Yuan Z, Cheng X, Duan Y. Impact of media dependence: how emotional interactions between users and chat robots affect human socialization? *Front Psychol* 2024; **15:** 1388860. https://doi.org/10.3389/fpsyg.2024.1388860

108 Zhang R, Li H, Meng H, Zhan J, Gan H, Lee Y-C. The Dark Side of AI Companionship: A Taxonomy of Harmful Algorithmic Behaviors in Human-AI Relationships. In: Yamashita N, Evers V, Yatani K, et al., eds. Proceedings of the 2025 CHI Conference on Human Factors in Computing Systems. New York, NY, USA: ACM, 2025: 1–17.

109 Zhang X, Yin M, Zhang M, Li Z, Li H. The Development and Validation of an Artificial Intelligence Chatbot Dependence Scale. *Cyberpsychol Behav Soc Netw* 2025; **28:** 126–31. https://doi.org/10.1089/cyber.2024.0240

110 Zhang X, Li H, Yin M, Zhang M, Li Z, Chen Z. Investigating AI Chatbot Dependence: Associations with Internet and Smartphone Dependence, Mental Health Outcomes, and the Moderating Role of Usage Purposes. *International Journal of Human–Computer Interaction* 2025: 1–13. https://doi.org/10.1080/10447318.2025.2545464

111 Zhang X, Li Z, Zhang M, et al. Exploring artificial intelligence (AI) Chatbot usage behaviors and their association with mental health outcomes in Chinese university students. *J Affect Disord* 2025; **380:** 394–400. https://doi.org/10.1016/j.jad.2025.03.141

112 Zhang K, Xie Y, Chen D, Ji Z, Wang J. Effects of attractions and social attributes on peoples' usage intention and media dependence towards chatbot: The

mediating role of parasocial interaction and emotional support. *BMC Psychol* 2025; **13:** 986. https://doi.org/10.1186/s40359-025-03284-w

113 Carlbring P, Andersson G. Commentary: AI psychosis is not a new threat: Lessons from media-induced delusions. *Internet Interv* 2025; **42:** 100882. https://doi.org/10.1016/j.invent.2025.100882

114 Chamarthi VS, Das P, Kashyap R. AI as a Novel Digital Stressor in Adolescent Psychosis: Clinical and Ethical Implications. *Int J Psychiatry Med* 2025: 912174251392768. https://doi.org/10.1177/00912174251392768

115 Hudon A, Stip E. Delusional Experiences Emerging From AI Chatbot Interactions or "AI Psychosis". *JMIR Ment Health* 2025; **12:** e85799. https://doi.org/10.2196/85799

116 Morrin H, Nicholls L, Levin M, et al. Delusions by design? How everyday AIs might be fuelling psychosis (and what can be done about it), 2025.

117 Osler L. Hallucinating with AI: AI Psychosis as Distributed Delusions. arXiv, 2025.

118 Østergaard SD. Will Generative Artificial Intelligence Chatbots Generate Delusions in Individuals Prone to Psychosis? *Schizophr Bull* 2023; **49:** 1418–19. https://doi.org/10.1093/schbul/sbad128

119 Yeung JA, Dalmasso J, Foschini L, Dobson RJB, Kraljevic Z. The Psychogenic Machine: Simulating AI Psychosis, Delusion Reinforcement and Harm Enablement in Large Language Models. arXiv, 2025.

Preferred Reporting Items for Systematic reviews and Meta-Analyses extension for Scoping Reviews (PRISMA-ScR) Checklist

SECTION	ITEM	PRISMA-ScR CHECKLIST ITEM	REPORTED ON PAGE #
**TITLE**			
Title	1	Identify the report as a scoping review.	Title
**ABSTRACT**			
Structured summary	2	Provide a structured summary that includes (as applicable): background, objectives, eligibility criteria, sources of evidence, charting methods, results, and conclusions that relate to the review questions and objectives.	no subheadings permitted
**INTRODUCTION**			
Rationale	3	Describe the rationale for the review in the context of what is already known. Explain why the review questions/objectives lend themselves to a scoping review approach.	5
Objectives	4	Provide an explicit statement of the questions and objectives being addressed with reference to their key elements (e.g., population or participants, concepts, and context) or other relevant key elements used to conceptualize the review questions and/or objectives.	6
**METHODS**			
Protocol and registration	5	Indicate whether a review protocol exists; state if and where it can be accessed (e.g., a Web address); and if available, provide registration information, including the registration number.	37 (available)
Eligibility criteria	6	Specify characteristics of the sources of evidence used as eligibility criteria (e.g., years considered, language, and publication status), and provide a rationale.	37-38
Information sources*	7	Describe all information sources in the search (e.g., databases with dates of coverage and contact with authors to identify additional sources), as well as the date the most recent search was executed.	38-39
Search	8	Present the full electronic search strategy for at least 1 database, including any limits used, such that it could be repeated.	Supplementary
Selection of sources of evidence†	9	State the process for selecting sources of evidence (i.e., screening and eligibility) included in the scoping review.	40
Data charting process‡	10	Describe the methods of charting data from the included sources of evidence (e.g., calibrated forms or forms that have been tested by the team before their use, and whether data charting was done independently or in duplicate) and any processes for obtaining and confirming data from investigators.	41
Data items	11	List and define all variables for which data were sought and any assumptions and simplifications made.	Supplementary
Critical appraisal of individual sources of evidence§	12	If done, provide a rationale for conducting a critical appraisal of included sources of evidence; describe the methods used and how this information was used in any data synthesis (if appropriate).	9
Synthesis of results	13	Describe the methods of handling and summarizing the data that were charted.	41

SECTION	ITEM	PRISMA-ScR CHECKLIST ITEM	REPORTED ON PAGE #
**RESULTS**			
Selection of sources of evidence	14	Give numbers of sources of evidence screened, assessed for eligibility, and included in the review, with reasons for exclusions at each stage, ideally using a flow diagram.	40
Characteristics of sources of evidence	15	For each source of evidence, present characteristics for which data were charted and provide the citations.	Supplementary
Critical appraisal within sources of evidence	16	If done, present data on critical appraisal of included sources of evidence (see item 12).	Supplementary
Results of individual sources of evidence	17	For each included source of evidence, present the relevant data that were charted that relate to the review questions and objectives.	8-29
Synthesis of results	18	Summarize and/or present the charting results as they relate to the review questions and objectives.	8-29
**DISCUSSION**			
Summary of evidence	19	Summarize the main results (including an overview of concepts, themes, and types of evidence available), link to the review questions and objectives, and consider the relevance to key groups.	29-35
Limitations	20	Discuss the limitations of the scoping review process.	36
Conclusions	21	Provide a general interpretation of the results with respect to the review questions and objectives, as well as potential implications and/or next steps.	37
**FUNDING**			
Funding	22	Describe sources of funding for the included sources of evidence, as well as sources of funding for the scoping review. Describe the role of the funders of the scoping review.	42

JBI = Joanna Briggs Institute; PRISMA-ScR = Preferred Reporting Items for Systematic reviews and Meta-Analyses extension for Scoping Reviews.
* Where *sources of evidence* (see second footnote) are compiled from, such as bibliographic databases, social media platforms, and Web sites.
† A more inclusive/heterogeneous term used to account for the different types of evidence or data sources (e.g., quantitative and/or qualitative research, expert opinion, and policy documents) that may be eligible in a scoping review as opposed to only studies. This is not to be confused with *information sources* (see first footnote).
‡ The frameworks by Arksey and O'Malley (6) and Levac and colleagues (7) and the JBI guidance (4, 5) refer to the process of data extraction in a scoping review as data charting.
§ The process of systematically examining research evidence to assess its validity, results, and relevance before using it to inform a decision. This term is used for items 12 and 19 instead of "risk of bias" (which is more applicable to systematic reviews of interventions) to include and acknowledge the various sources of evidence that may be used in a scoping review (e.g., quantitative and/or qualitative research, expert opinion, and policy document).

From: Tricco AC, Lillie E, Zarin W, O'Brien KK, Colquhoun H, Levac D, et al. PRISMA Extension for Scoping Reviews (PRISMA-ScR): Checklist and Explanation. Ann Intern Med. ;169:467–473. doi: 10.7326/M18-0850
